# The Triterpenoid CDDO-Methyl Ester Reduces Tumor Burden, Reprograms the Immune Microenvironment, and Protects from Chemotherapy-Induced Toxicity in a Preclinical Mouse Model of Established Lung Cancer

**DOI:** 10.3390/antiox13060621

**Published:** 2024-05-21

**Authors:** Jessica A. Moerland, Karen T. Liby

**Affiliations:** 1 Department of Pharmacology and Toxicology, Michigan State University, 1355 Bogue Street, East Lansing, MI 48824, USA; moerlan2@msu.edu; 2 Division of Hematology/Oncology, Department of Medicine, Indiana University School of Medicine, 980 W. Walnut Street, Indianapolis, IN 46202, USA

**Keywords:** CDDO-Methyl ester, triterpenoid, lung cancer, tumor microenvironment, immunomodulation, chemotherapy, carboplatin, paclitaxel

## Abstract

NRF2 activation protects epithelial cells from malignancy, but cancer cells can upregulate the pathway to promote survival. NRF2 activators including CDDO-Methyl ester (CDDO-Me) inhibit cancer in preclinical models, suggesting NRF2 activation in other cell types may promote anti-tumor activity. However, the immunomodulatory effects of NRF2 activation remain poorly understood in the context of cancer. To test CDDO-Me in a murine model of established lung cancer, tumor-bearing wildtype (WT) and Nrf2 knockout (KO) mice were treated with 50–100 mg CDDO-Me/kg diet, alone or combined with carboplatin/paclitaxel (C/P) for 8–12 weeks. CDDO-Me decreased tumor burden in an Nrf2-dependent manner. The combination of CDDO-Me plus C/P was significantly (*p* < 0.05) more effective than either drug alone, reducing tumor burden by 84% in WT mice. CDDO-Me reduced the histopathological grade of WT tumors, with a significantly (*p* < 0.05) higher proportion of low-grade tumors and a lower proportion of high-grade tumors. These changes were augmented by combination with C/P. CDDO-Me also protected WT mice from C/P-induced toxicity and improved macrophage and T cell phenotypes in WT mice, reducing the expression of CD206 and PD-L1 on macrophages, decreasing immunosuppressive FoxP3+ CD4+ T cells, and increasing activation of CD8+ T cells in a Nrf2-dependent manner.

## 1. Introduction

Lung cancer remains the leading cause of cancer-related deaths in the United States and the most frequently diagnosed cancer in both men and women [1]. For many patients, the paradigm of first-line therapy for advanced disease has shifted towards immune checkpoint blockade, alone or in combination with chemotherapy [2], and this change has transformed the clinical management of non-small cell lung cancer. Patients who respond to immunotherapy have profound, durable improvements which lead to reduced mortality in patients [3]. However, less than 30% of patients respond to immunotherapies, and the identification of predictive biomarkers of patient response remains a challenge [4]. Targeted therapies often have limited efficacy and multiple adverse effects, and the predicament of drug resistance has severely restricted the long-term survival advantage for novel agents including *KRAS^G12C^* inhibitors [5]. Therefore, the identification of novel therapeutic agents and elucidation of mechanisms driving disease pathogenesis are critical to improving clinical outcomes for lung cancer.

The success of immune checkpoint inhibitors in responsive patients illustrates the anti-tumor potential of the immune microenvironment in the lung. In many established untreated lung cancers, the immune compartment suppresses an anti-tumor response and promotes pro-tumor immune activity [6,7]. Anti-tumor immune responses produce a harsh, inflammatory environment characterized by hypoxia and abundant oxidative stress [8,9,10]. While tumor cells exploit endogenous mechanisms to tolerate the high levels of stress in the lung tumor microenvironment, immune cells have lower antioxidant capacity and are more sensitive to oxidative damage, resulting in cellular dysfunction and death [11,12,13]. The nuclear factor erythroid 2-related factor 2 (NRF2)-KEAP1-ARE signaling axis is a master regulator of antioxidant defenses [14,15]. Up to 26% of human lung adenocarcinomas acquire mutations resulting in constitutive NRF2 activation, which promotes tumor cell survival. However, constitutive activation of the NRF2 pathway is absent in the immune compartment, and these cells remain vulnerable to oxidative stress [16]. In order to establish and maintain a pro-inflammatory anti-tumor response, immune cells are dependent on cytoprotective mechanisms including NRF2 activation [17].

The tumor suppressive effects of NRF2 activation in immune cells in lung cancer, particularly in the myeloid lineage [18,19], were demonstrated. Macrophages make up a significant portion of the immune microenvironment in lung tumors, and their dynamic ability to fluctuate between anti-tumor and tumor-promoting phenotypes makes these cells an attractive target for anti-cancer therapies [20]. However, most previous studies evaluating NRF2 activation in macrophages within the lung tumor microenvironment utilized models of early-stage lung cancer, and NRF2 plays differing roles throughout carcinogenesis [21]. In non-transformed cells, NRF2 activation protects against malignant transformation, but increased NRF2 activity in cancer cells promotes survival and tumor progression [22]. The effects of pharmacological NRF2 activation on immune cells in the microenvironment of established lung cancer have not been fully elucidated. Oleanane triterpenoids activate the NRF2 pathway with potency and selectivity at nanomolar concentrations [23], and the anti-tumor effects of these drugs have been demonstrated in murine models for both cancer prevention and treatment [19,24,25,26]. However, the effects of CDDO-Methyl ester (CDDO-Me or bardoxolone methyl) on immune cells in the lung tumor microenvironment remain underexplored, particularly in the advanced stages of the disease. Because most lung cancers are not diagnosed until they reach the later stages of III/IV [1], it is critical to evaluate the effects of NRF2 activation on immune cells in models of established lung cancer and not just for prevention.

Despite advancements in lung cancer treatment options, cytotoxic chemotherapy remains a critical component of the clinical standard of care for this disease. However, the adverse effects of these drugs induce widespread multiorgan toxicities including decreased white blood cell count, hair loss, gastrointestinal effects, and weight loss [27]. These characteristics adversely affect patient quality of life and may worsen existing underlying health challenges. While CDDO-Me enhances the anti-tumor efficacy of the chemotherapeutic agents carboplatin and paclitaxel (C/P), demonstrated by a significantly greater reduction in tumor burden with the combination of CDDO-Me + C/P than either agent alone [24], how the combination of these drugs affects the immune microenvironment is unknown. Additionally, the specific role of NRF2 in the anti-tumor response has not been delineated. We hypothesized that CDDO-Me induces changes to the lung tumor immune microenvironment and that the anti-tumor effects of CDDO-Me are dependent on Nrf2. Because immune cells are particularly susceptible to both oxidative stress and chemotherapy-induced toxicity, combining CDDO-Me and chemotherapy should favorably modulate the activity of immune cells while simultaneously protecting them from toxicity, facilitating anti-tumor immunity and augmenting the efficacy of chemotherapy.

To test our hypotheses, wildtype (WT) and Nrf2 knockout (KO) A/J mice with established lung tumors were treated with the triterpenoid CDDO-Me, alone and in combination with the cytotoxic chemotherapeutic drugs carboplatin and paclitaxel. These drugs are commonly used clinically for lung cancer treatment because of their distinct mechanisms and efficacy. Carboplatin crosslinks DNA, thereby disrupting DNA replication, while paclitaxel prevents cell division by inhibiting microtubule depolymerization. Combining paclitaxel with carboplatin significantly increases DNA adduct formation, making this specific cocktail more effective in blocking tumor cell proliferation than either agent alone [28].

A/J mice are sensitive to the carcinogen vinyl carbamate, a mutagenic metabolite of urethane found in tobacco smoke [29]. Vinyl carbamate induces *Kras* mutations and subsequent aggressive lung adenocarcinomas, making this an experimental model of high clinical relevance as adenocarcinomas account for at least half of all human lung cancer cases [1]. To characterize the immunomodulatory effects of CDDO-Me ± C/P, lungs were processed for flow cytometry using an optimized immunophenotyping panel [30]. To determine the necessity of Nrf2 for the anti-tumor effects of CDDO-Me, Nrf2 KO A/J mice were used in addition to WT A/J mice.

## 2. Materials and Methods

**Treatment of lung adenocarcinomas in vivo**

All mouse studies were performed in accordance with AAALAC-accredited Standards for the Management of Laboratory Animals and an animal protocol (202100188) approved by the Institutional Animal Care and Use Committee at Michigan State University. A/J mice are widely used to model lung cancer and for carcinogen testing given their high susceptibility to carcinogen-induced tumors. WT A/J mice were originally obtained from Jackson Laboratories, and Nrf2 KO A/J mice were genetically engineered by CRISPR-Cas9 editing of the *Nrf2* locus ENSMUSG00000015839 (Accession # NM_010902.5) as described [19]; mice of both genotypes were bred in-house.

Between 6 and 7 weeks of age, male and female WT and Nrf2 KO A/J mice were injected i.p. with 0.32 mg vinyl carbamate (Toronto Research Chemicals #TRC-V375000; PubChem CID: 27492) per mouse. Mice were injected with a second dose of vinyl carbamate one week later. Beginning one week before initiation with vinyl carbamate, mice were fed a well-characterized semi-synthetic AIN-93G rodent diet (BioServ, Flemington, NJ, USA) plus 12.5 mL ethanol and 37.5 mL Neobee oil (Spectrum Chemical, New Brunswick, NJ, USA) per kg diet. The control diet was continued for an additional 8 weeks to allow tumors to develop, after which mice were randomized into treatment groups. The number of mice per group was determined based on variability observed in previous studies evaluating CDDO-Me in A/J mice [19].

Diets were prepared by dissolving CDDO-Me (treatment) into 12.5 mL ethanol and 37.5 mL Neobee oil (vehicle) and then mixing the liquids into one kg AIN-93G powder diet for 20 min using a KitchenAid mixer. After allowing tumors to grow for 8 weeks, mice were fed a treatment diet (50–100 mg CDDO-Me/kg AIN-93G, ~12.5–25 mg/kg body weight) intermittently (one week on CDDO-Me diet, followed by one week on control diet) for an additional 8 weeks (Study 1).

In Study 2, mice were initiated with vinyl carbamate as described and tumors allowed to grow for 8 weeks before being treated with CDDO-Me (80 mg/kg or ~20 mg/kg body weight), alone and in combination with carboplatin (50 mg/kg) (Sigma-Aldrich, St. Louis, MO, USA) and paclitaxel (15 mg/kg) (Cayman Chemical, Ann Arbor, MI, USA) (C/P). Six total doses of C/P were injected i.p. on the same day every other week over the longer 12-week treatment period. Mice not treated with C/P were injected i.p. with the vehicle for these drugs (DMSO:cremophor:saline 1:1:8) on the same days as the mice receiving C/P as an additional control.

After 8–12 weeks on the treatment diet, the blood and lungs were harvested. The lungs were dissected and inflated with PBS for quantification of grossly visible tumors on the surface of the lungs. The left lung was fixed in 10% neutral-buffered formalin for 48 h and then embedded in paraffin. The lung was step-sectioned: the first section from 200 microns deep in the embedded tissue and a second section after an additional 800 microns. Both sections were then stained with hematoxylin and eosin for grading. Sample group identities were blinded and then slides were randomized. Tumors were counted on each slide, measured, and graded for histopathological severity as previously described [19]. Samples were then unblinded and data were summarized for statistical analysis. Additional unstained sections were used for the immunohistochemistry of biomarkers. The right lobes of the lung were divided and either analyzed using flow cytometry or flash-frozen and stored at −80 °C for western blotting.

**Western blotting**

Flash-frozen lung tissues from the right lung were homogenized in EBC buffer containing protease inhibitors (aprotinin, PMSF, and leupeptin) and 10% NP-40. Samples were incubated on ice for 40 min with agitation and vortexing followed by sonication every 10 min. Protein concentrations were determined using the BCA assay (Sigma-Aldrich, St. Louis, MO, USA) and concentrations were normalized in resolving SDS-PAGE buffer. Samples were separated through 10% polyacrylamide gels at 100 V and proteins were transferred onto a nitrocellulose membrane at 100 V for 2.5 h. Membranes were probed with antibodies against NQO1 (1:1000, 5% BSA, Invitrogen #PA5-115666, Waltham, MA, USA) and β-actin (1:1000, 5% BSA, Sigma-Aldrich #A-5441), followed by rabbit and mouse secondary fluorescent antibodies (1:1000, 5% BSA, LI-CORE #926-32213 and #926-32212, respectively). Membranes were dried then visualized using the BioRad ChemiDoc imaging system and quantified using FIJI 1.0 software.

**Immunohistochemical or immunofluorescent staining**

Sections of the left lobe from 1000 microns deep were obtained from formalin-fixed, paraffin-embedded mouse lungs and mounted. Sections were immunostained for NQO1 (1:100, Invitrogen #PA5-115666), PCNA (1:100, Santa Cruz Biotechnology #SC-56, Dallas, TX, USA), and p-ERK (1:200, Cell Signaling #4370, Danvers, MA, USA) and visualized using HRP-conjugated secondary antibodies (αRat 1:1000, Vector Laboratories #BA-9400, Newark, CA, USA and αRabbit 1:1000, Cell Signaling #8114S, respectively). Slides were counterstained with hematoxylin and visualized at 40× magnification by light microscopy. For immunofluorescence, sections were TUNEL stained using a commercially available kit (Invitrogen #C10618). Slides were counterstained with DAPI and imaged on an Invitrogen EVOS M5000 digital color fluorescence microscope equipped with DAPI and Texas Red filters.

**Flow cytometry**

The same two lobes of the right lung were harvested from female A/J mice (n = 4–10 mice/group) and incubated in digestion media containing collagenase (300 U/mL, Sigma-Aldrich) and DNAse (2 U/mL, Calbiochem) in DMEM (Corning, Corning, NY, USA) for 30 min at 37 °C with stirring. Samples were then passed through a 40 µm cell strainer (ThermoFisher, Waltham, MA, USA) to break apart cell clumps, and red blood cells were eliminated using lysis solution. Fc receptors were blocked using 5 μg/mL anti-mouse CD16/CD32 for 10 min on ice. Single cells were resuspended in a 1:1 solution of Brilliant Violet buffer (BD Bioscience, Franklin Lakes, NJ, USA):PBA and stained for 30 min on ice using an optimized antibody panel [30] for immune cell targets including αCD45 (BioLegend, San Diego, CA, USA, BV510 #103138), αCD11b (BioLegend PE/Cyanine7 #101216), αCD11c (BioLegend PE/Cyanine5 #117316), αCD64 (BioLegend BV711 #139311), αCD206 (BioLegend BV421 #141717), αPD-L1 (BioLegend PE-Dazzle594 #124324), αCD4 (Miltenyi Biotrc FITC, Gaithersburg, MD, USA, #130-120-819), αCD8 (Invitrogen BUV805 #368-0081-82), αCD25 (BioLegend PE #101904), αCD24 (BioLegend BV605 #101827), and αCD107a (BioLegend Alexa Fluor 647 #121610). Cells were stained for viability with Live/Dead Fixable Blue Dead Cell Stain Kit (Invitrogen #L23105). Cells were permeabilized using the BD Biosciences Cytofix/Cytoperm Fixation/Permeabilization kit (#554714) and stained for the intracellular target αFoxP3 (Invitrogen Alexa Fluor 700 #56-5773-80). Cell samples were run on a Cytek Aurora spectral flow cytometer equipped with 5 lasers (UV 355 nm, violet 405 nm, blue 488 nm, yellow-green 561 nm, and red 640 nm). Data were analyzed using FlowJo x.10.0.7r2 software using an optimized gating strategy [30].

**Isolation of T cells from spleen**

The 6–7-week-old female A/J mice (WT and Nrf2 KO) were euthanized according to IACUC guidelines. The spleen was harvested, and individual cells were released by compressing between two frosted glass slides. CD4+ T cells were isolated using a commercial magnetic column separation system (Miltenyi Biotec, Gaithersburg, MD, USA) and activated with αCD28 (3 μg/mL), IL-2 (5 ng/mL), and TGF-β (5 ng/mL) for 24 h. Cells were treated with vehicle (DMSO) or CDDO-Me (10 nM) for an additional 24 h and analyzed by qPCR.

**qPCR**

RNA was isolated using TRIzol (Invitrogen). A total of 2 μg RNA was used to synthesize cDNA utilizing the SuperScript III reverse transcriptase kit (Invitrogen) and the following conditions: 10 min 25 °C, 2 h 37 °C, 5 min 85 °C, indefinite hold at 4 °C. Primer (IDT) sequences were as follows: FoxP3 (F 5′-CTCGTCTGAAGGCAGAGTCA-3′ & R 5′-TGGCAGAGAGGTATTGAGGG-3′) and RPL-13a (F 5′-GTTGATGCCTTCACAGCGTA-3′ & R 5′-AGATGGCGGAGGTGCAG-3′). An optimized qPCR protocol was run using a QuantStudio 7 Flex Real-Time PCR machine: 2 min 50 °C; 10 min 95 °C; 15 s 95 °C; 1 min 60 °C; repeat steps 3–4 40×; 15 s 95 °C; 1 min 60 °C. Fold change in target RNA expression was quantified utilizing the ∆∆CT method.

**Complete blood counts (CBCs)**

Immediately after the euthanasia of mice, blood was collected via cardiac puncture with a 25 g needle attached to a 1 mL syringe pre-filled with 50 μL EDTA into a K2 EDTA tube. Complete blood counts were obtained using an Idexx ProCyte Dx hematology analyzer.

**Statistics**

A statistical analysis of data was completed using either GraphPad Prism 10 or SigmaStat 3.5 software. The following tests were used: two-way ANOVA followed by Tukey HSD for multiple comparisons for in vitro mRNA transcripts in CD4+ splenocytes and for in vivo comparisons of more than two groups, and a z-test for comparing proportions of mouse mortality and tumor histopathology. All in vitro studies were repeated at least three times, and representative results were shown. For all studies, an alpha value of α = 0.05 was used for statistical significance.

## 3. Results

### 3.1. Study 1: CDDO-Me Reduces Tumor Burden and Histopathological Severity of Established Lung Tumors in a Dose- and Nrf2-Dependent Manner

**Figure 1:**
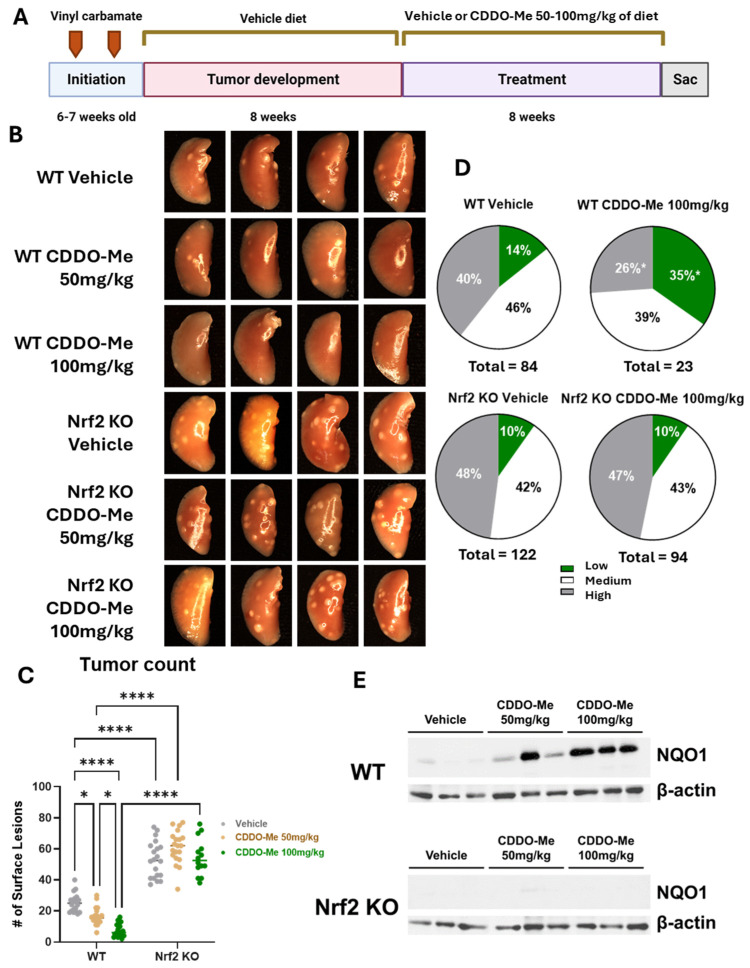
**CDDO-Methyl ester (CDDO-Me) reduces tumor burden and the severity of tumor histopathology of established lung tumors in a dose- and Nrf2-dependent manner**. (**A**) Schematic detailing experimental design for Study 1. Mice were challenged with vinyl carbamate and fed a vehicle (ethanol + Neobee oil) diet while lung tumors developed, after which mice were randomized into groups fed either a vehicle diet or a diet containing 50–100 mg/kg CDDO-Me dissolved in the vehicle. CDDO-Me was dosed intermittently (one week on the CDDO-Me diet followed by one week on the vehicle diet). (**B**) Representative images (4 mice/group, 8× magnification) of the left lung of wildtype (WT) and Nrf2 knockout (KO) mice at endpoint. (**C**) Quantitation of surface tumors on both right and left lungs of study mice (n = 12–18 mice/group). (**D**) Proportions of total lung tumors on lung sections for each histopathological grade. (**E**) Western blot of NQO1 and β-actin proteins isolated from lung homogenates (n = 3 mice/treatment group). Statistics: Two-way ANOVA followed by Tukey HSD (**C**); * *p* < 0.05 or **** *p* < 0.0001 as shown in the panel. Z test for proportions (**D**); * *p* < 0.05 vs. WT Vehicle.

Both wild-type (WT) and Nrf2 knockout (KO) mice on an A/J background were challenged with vinyl carbamate to initiate lung cancer. Tumors were allowed to grow for an additional eight weeks, which is sufficient to allow the detection of tumors within the lung [24]. Mice were then randomized into treatment groups and fed a diet containing either vehicle control or 50–100 mg of CDDO-Me/kg diet intermittently (one week on CDDO-Me diet, one week on the control diet) for an additional eight weeks (Figure 1A). These concentrations equate to ~12.5–25 mg/kg of total body weight, based on the average amount of food consumed each day [25]. These concentrations were the same as previous studies demonstrating the anti-tumor efficacy of CDDO-Me in WT A/J mice challenged with vinyl carbamate in later-stage intervention studies [24]. The mice tolerated this treatment well, as they continued to gain weight throughout the study at a rate equivalent to mice receiving the vehicle diet, and no significant differences in weight were found in any groups (Figure S1).

Upon gross observation of the lungs, the differences in surface tumor numbers between groups were striking (Figure 1B). When these surface tumors were quantified, a significant dose-response was observed in WT mice (Figure 1C). WT mice treated with 50 mg/kg CDDO-Me had 34.4% fewer (*p* < 0.05) average surface tumors compared to WT mice on vehicle diet, with averages of 16.5 ± 1.3 tumors per mouse in the treated group compared to 25.1 ± 1.3 tumors in the vehicle group. The 100 mg/kg dose of CDDO-Me was even more effective, lowering (*p* < 0.0001) average surface tumor count from 22.4 ± 1.3 in the vehicle group to 6.9 ± 1.0, a reduction of nearly 70% (Table 1A,B). The average surface tumor count in Nrf2 KO mice was nearly 2.5-fold (*p* < 0.0001) higher compared to WT mice with an average of 62.5 ± 2.6 tumors per mouse (Table 1A,B and Figure 1C), and this striking increase can be appreciated in the lung images displayed in Figure 1B. Importantly, no differences in tumor count were observed in the Nrf2 KO mice, regardless of treatment, suggesting the dose-dependent anti-tumor effects of CDDO-Me are Nrf2 dependent.

When lung sections were evaluated using microscopy, treatment with 50 mg/kg CDDO-Me reduced tumor size and burden in WT mice by 54.8% and 67.4%, respectively, compared to WT mice on a vehicle diet. Tumors in the vehicle group averaged a size of 0.26 ± 0.08 mm^3^ and a burden of 0.57 ± 0.3 mm^3^ compared to 0.12 ± 0.05 mm^3^ and 0.19 ± 0.3 mm^3^, respectively, in mice treated with 50 mg/kg CDDO-Me. CDDO-Me at 100 mg/kg was even more effective than the lower dose, as it reduced average tumor size and burden (*p* < 0.001) by 60.3% and 89.1%, respectively (Table 1). This dose-dependent change complemented the improvements in surface tumor counts. Additionally, CDDO-Me at 100 mg/kg significantly (*p* < 0.05) reduced tumor grade by increasing the proportion of the least aggressive low-grade tumors while simultaneously decreasing (*p* < 0.05) the proportion of the more aggressive high-grade tumors (Figure 1D and Table 1B). While only 14.3% of all tumors in WT mice receiving vehicle diet were the least aggressive low-grade phenotype, this proportion was 34.8% (*p* < 0.05) in the lungs of mice treated with 100 mg/kg CDDO-Me. This change was complemented by a decrease (*p* < 0.05) in the proportion of the aggressive, high-grade tumor phenotype from 39.3% in the vehicle group to 26.1% of all tumors in the group treated with 100 mg/kg of CDDO-Me. Importantly, the lower dose of 50 mg/kg CDDO-Me did not alter tumor histopathology in WT mice, illustrating a dose-dependent effect of CDDO-Me on tumor histopathology in which the lower dose of 50 mg/kg was insufficient to improve tumor histopathological grade (Table 1A and Figure S2). In contrast, Nrf2 KO mice fed CDDO-Me had no reduction in tumor size, number, or overall burden, and there was no alteration of histopathological grade by either dose, demonstrating Nrf2-dependent anti-tumor efficacy (Figure 1 and Table 1). Activation of the Nrf2 pathway in the lungs of WT mice, but not Nrf2 KO mice, treated with CDDO-Me was confirmed by western blot by an increase in NQO1, a downstream target of Nrf2, expression in protein isolated from lung homogenates (Figure 1E). Because Nrf2 is constitutively synthesized and degraded, changes in Nrf2 expression levels may not correlate with activation of the pathway [31]. NQO1 is frequently used as a surrogate marker of Nrf2 activation, as expression of this protein is regulated almost exclusively by Nrf2 [32].

### 3.2. CDDO-Me Increases NQO1 Expression in the Lungs of WT but Not Nrf2 KO Mice and Modulates Activity but Not Infiltration of Macrophages and T Cells

**Figure 2:**
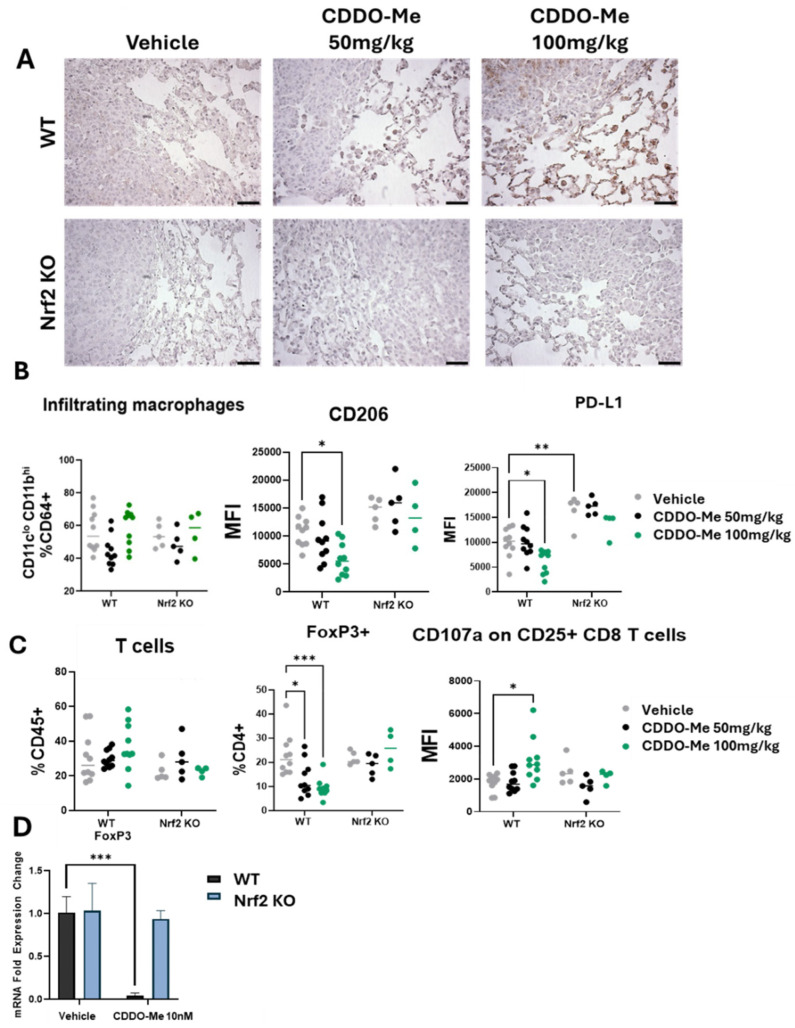
**CDDO-Me increases NQO1 staining in the lungs of WT mice and modulates the phenotype but not infiltration of macrophages and T cells**. (**A**) Immunohistochemical staining of NQO1 in the lungs of WT and Nrf2 KO mice treated with 50–100 mg/kg CDDO-Me. Scale bar = 60 microns. (**B**,**C**) Flow cytometry analysis of CD45^+^ cells in the lungs of WT and Nrf2 KO mice at the study endpoint as described in Figure 1A. Flow cytometry analysis of infiltrating macrophages (CD45^+^ CD11c^lo^ CD11b^hi^ CD64^+^ cells, % CD64^+^) and mean fluorescence intensity of CD206 and PD-L1 on infiltrating macrophages (**B**) or total T cells (% CD45^+^), FoxP3^+^ CD4^+^ T cells (% CD4^+^), and mean fluorescence intensity (MFI) of CD107a on CD45^+^ CD25^+^ CD8^+^ cells (**C**) in the lung. (**D**) Fold expression change of FoxP3 mRNA in CD4+ splenocytes isolated from WT and Nrf2 KO mice and activated with αCD28, IL-2, and TGF-β and then treated with vehicle or 10 nM CDDO-Me for 24 h. Statistics: Two-way ANOVA followed by Tukey HSD. * *p* < 0.05; ** *p* < 0.01; *** *p* < 0.001.

To further evaluate Nrf2 activation levels in the lungs of mice treated with CDDO-Me, the Nrf2 target NQO1 was analyzed by immunohistochemistry. Importantly, CDDO-Me increased NQO1 staining in both the tumor and in the cells within the surrounding microenvironment in WT mice (Figure 2A). In contrast, NQO1 was not expressed in the lungs of Nrf2 KO mice on vehicle or CDDO-Me treatment. To detect changes in immune cell populations within the lung tumor microenvironment, lungs were digested for flow cytometry, and immune cells were evaluated using CD45 as a pan-immune cell marker. The total number of macrophages and T cells in the lungs were unchanged (Figure 2B,C). Importantly, expression of the tumor-promoting macrophage marker CD206 was decreased (*p* < 0.05) in the lungs of WT mice fed the high dose (100 mg/kg diet) of CDDO-Me (Figure 2B). While a slight reduction in CD206 expression was observed in WT mice treated with 50 mg/kg CDDO-Me, this trend did not reach statistical significance, again demonstrating the dose-dependent effects of CDDO-Me (Figure 2B). CD206 is a well-characterized marker of pro-tumor macrophages, and a higher proportion of this macrophage subtype in the lungs of cancer patients correlates with poor prognosis and reduced overall survival [33]. The immunosuppressive marker PD-L1 also was lower (*p* < 0.05) on infiltrating macrophages at the same dose of CDDO-Me in WT mice but was significantly higher (*p* < 0.01) in Nrf2 KO mice compared to WT mice, regardless of treatment (Figure 2B).

PD-L1 regulates the anti-tumor T cell response [34] and indeed, CDDO-Me favorably altered both CD4+ and CD8+ T cell populations in the lungs of WT mice. Clinically, PD-L1 expression is mostly evaluated in tumor cells, but increased expression on macrophages within the tumor microenvironment has the same immunosuppressive effect on T cells [35]. FoxP3, a marker of immunosuppressive regulatory T cells that also correlates with poor patient prognosis [36], was decreased in WT mice treated with CDDO-Me in a dose-dependent manner (Figure 2C). Additionally, expression of the degranulation marker CD107a on CD25+ CD8+ T cells, a biomarker of activated cytotoxic anti-tumor CD8+ T cells [37], was higher in WT mice treated with 100 mg/kg CDDO-Me (Figure 2C). Importantly, CDDO-Me did not alter the expression of CD206, PD-L1, FoxP3, or CD107a in the Nrf2 KO mice, intriguingly showcasing the necessity of Nrf2 for these immunomodulatory effects.

The favorable decrease in FoxP3 expression on CD4+ T cells was also observed in vitro. Splenocytes isolated from WT A/J mice and activated by a cocktail of αCD28, IL-2, and TGF-β expressed lower (*p* < 0.001) FoxP3 mRNA transcripts when treated with 10 nM CDDO-Me (Figure 2D). Importantly, no changes in immune phenotypes, analyzed either by flow cytometry in vivo or by qPCR in vitro, were observed in Nrf2 KO mice treated with CDDO-Me, strongly suggesting a dependence on Nrf2 for these phenotypic changes.

### 3.3. Study 2: CDDO-Me, Alone and in Combination with Carboplatin and Paclitaxel, Reduces Lung Tumor Burden and the Severity of Tumor Histopathology in a Nrf2-Specific Manner

**Figure 3:**
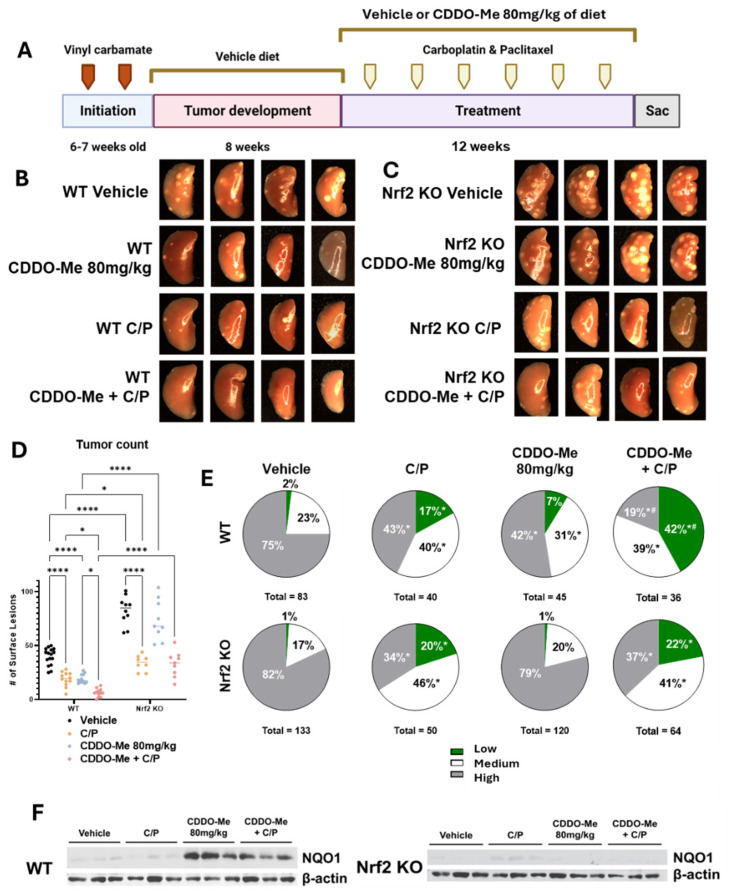
**CDDO-Me, alone and in combination with carboplatin and paclitaxel, reduces lung tumor burden and severity of tumor histopathology of advanced lung tumors in a dose- and Nrf2-dependent manner**. (**A**) Schematic detailing experimental design for Study 2: WT and Nrf2 KO mice were challenged with vinyl carbamate and fed a vehicle (ethanol + Neobee oil) diet for 8 weeks while tumors developed in the lung, after which they were randomized into treatment groups. Mice were fed either a vehicle diet or a diet containing 80 mg/kg CDDO-Me. The combination group was also injected with carboplatin and paclitaxel (C/P) at 50 mg/kg and 15 mg/kg of body weight, respectively. C/P was dosed i.p. once every other week on the same day for a total of 6 doses. Representative images (4 mice/group, 8×) of the left lung of wildtype mice (**B**) or Nrf2 knockout mice (**C**) at endpoint. (**D**) Quantification of surface tumors on both right and left lungs of mice (n = 7–16 mice/group). (**E**) Proportions of total lung tumors on lung sections for each histopathological grade. (**F**) Western blot of proteins isolated from whole lung homogenate (n = 3 animals/treatment group). Statistics: Two-way ANOVA followed by Tukey HSD (**D**): * *p* < 0.05 or **** *p* < 0.0001 as shown in the panel. Z test for proportions (**E**): * *p* < 0.05 vs. WT Vehicle; ^#^ *p* < 0.05 vs. WT C/P and WT CDDO-Me + C/P.

Triterpenoids can enhance efficacy and decrease toxicity induced by cytotoxic agents [24], but the necessity of Nrf2 activation and the effects on immune cells by this combination treatment was not known. To evaluate changes in the immune microenvironment with the combination of CDDO-Me and chemotherapy, lung tumors in WT and Nrf2 KO A/J mice challenged with vinyl carbamate were allowed to grow for 8 weeks. In this second study (Table 2), mice were randomized and treated with either a vehicle control diet or a diet containing 80 mg/kg CDDO-Me (20 mg/kg of body weight, intermittent with control diet) with or without chemotherapy. Six doses of carboplatin and paclitaxel (C/P) were injected intraperitoneally every other week at 50 mg/kg and 15 mg/kg, respectively, over 12 weeks of treatment (Figure 3A), 4 weeks longer than Study 1 (Figure 1, Table 1). The differences in the number of surface tumors between groups are remarkably evident in the images shown in Figure 3B,C. Not only were CDDO-Me and C/P effective at reducing tumor burden as single agents in the WT mice, but surface tumors were almost entirely absent in WT mice that received the combination treatment (Figure 3D). Quantification of the lungs revealed the impressive efficacy of the treatments both as single agents and in combination. C/P and CDDO-Me reduced the average number of surface tumors from 39.9 ± 2.0 in the vehicle group to 18.9 ± 1.9 and 21 ± 1.2, respectively, and the combination further reduced the average surface tumor count to 5.8 ± 1.1 tumors per mouse, a reduction of 85.5% (Figure 3D and Table 2). While the average surface tumor count was more than two-fold higher (*p* < 0.0001) in Nrf2 KO than WT mice, C/P reduced (*p* < 0.0001) surface tumor counts in Nrf2 KO mice by 57.8%, from an average of 91.8 ± 4.2 tumors per Nrf2 KO mouse in the vehicle group to an average of 38.7 ± 2.6 tumors per Nrf2 KO mouse treated with C/P. Interestingly, treatment with CDDO-Me alone in the Nrf2 KO mice decreased surface tumor count by 19.6%, suggesting an Nrf2-independent effect. Because the mechanism of CDDO-Me involves interaction with a cysteine residue on Keap1, the negative regulator of Nrf2, Nrf2 is not the lone target of CDDO-Me and these effects are likely due to a secondary target of the drug [23]. Notably, interaction with cysteine residues is a common mechanism of protein modification and is not unique to Keap1. However, the effects of CDDO-Me in the WT mice were strikingly more substantial than the comparatively small differences observed in the Nrf2 KO mice, suggesting that the Nrf2-dependent effect of CDDO-Me is distinct from the Nrf2-independent effects. Additionally, anti-tumor mechanisms of C/P are Nrf2-independent, as these drugs were effective in the Nrf2 KO mice (Figure 3B,C).

Similar to the changes in surface lesions, CDDO-Me and C/P reduced (*p* < 0.0001) tumor size and burden as single agents as evident in sectioned lungs of WT mice. The combination was significantly more effective at reducing tumor burden compared to either agent alone, decreasing the average tumor burden by 93.4% compared to WT mice on the vehicle. Tumor size and burden in WT mice on the vehicle treatment averaged 0.42 ± 0.16 mm^3^ and 1.09 ± 0.25 mm^3^, respectively, while the size and burden of tumors present in WT mice receiving CDDO-Me plus C/P averaged 0.05 ± 0.02 mm^3^ and 0.07 ± 0.2 mm^3^, respectively (Table 2). Nrf2 KO mice on the vehicle treatment had a nearly 7-fold higher (*p* < 0.0001) average tumor burden compared to WT mice (7.17 ± 1.2 mm^3^ vs. 1.09 ± 0.25 mm^3^), and treatment with C/P reduced average tumor size and burden in Nrf2 KO mice by 80.4% (0.19 ± 0.09 mm^3^) and 90.7% (0.67 ± 0.18 mm^3^), respectively (Table 2), compared to the Nrf2 KO controls. Consistent with changes observed with CDDO-Me alone on surface tumor counts, a 20.8% reduction in tumor burden was also observed in Nrf2 KO mice treated with CDDO-Me (Table 2) compared to the Nrf2 vehicle group, again suggesting secondary anti-tumor mechanisms of CDDO-Me, which are independent of Nrf2.

Distinct differences in tumor histopathological grades were also observed. The proportion of the less aggressive low- and medium-grade tumors was significantly (*p* < 0.05) higher in both WT and Nrf2 KO mice treated with C/P (Figure 3E and Table 2). As a single agent, treatment with CDDO-Me alone decreased the proportion of high-grade tumors and increased the proportion of medium-grade tumors in WT mice (Figure 3E and Table 2). Strikingly, the proportion of the least aggressive low-grade tumors was 41.7% (*p* < 0.05) in mice treated with the combination of CDDO-Me and C/P compared to only 2.4% of tumors in WT mice on vehicle diet. The proportion of high-grade tumors was also lower in the WT combination treatment group than in the control group, 19% vs. 75%, respectively. This decreased histopathological severity was significantly (*p* < 0.05) better in WT mice on the combination treatment compared to either CDDO-Me or C/P alone (Figure 3E and Table 2). In contrast to its striking efficacy in WT mice, CDDO-Me had no effect on tumor histopathological grades in the Nrf2 KO mice, consistent with an Nrf2-dependent effect. NQO1 protein was detected in lung homogenates from WT but not Nrf2 KO mice fed CDDO-Me, confirming Nrf2 pathway activation in vivo (Figure 3F).

**Figure 4:**
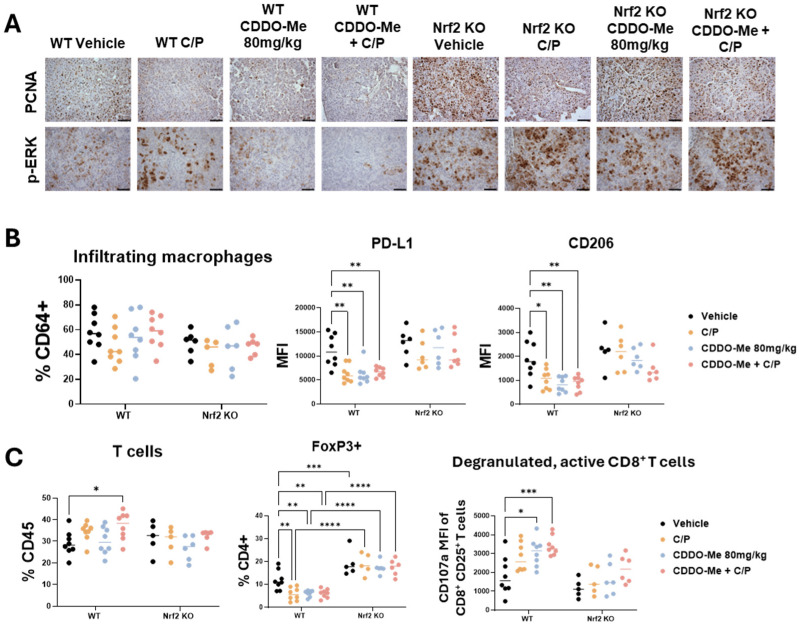
**CDDO-Me modulates macrophage and T cell phenotype in a Nrf2-dependent manner.** Immunohistochemical staining (**A**) of PCNA and p-ERK in lung sections from wildtype (WT) and Nrf2 knockout (KO) mice treated with vehicle, carboplatin/paclitaxel (C/P), CDDO-Me 80 mg/kg, or the combination as shown in Figure 3A; scale bar represents 60 μm. Flow cytometry analysis of infiltrating macrophages (CD45^+^ CD11c^lo^ CD11b^hi^ CD64^+^ cells, % CD64^+^) and mean fluorescence intensity (MFI) of CD206 and PD-L1 on infiltrating macrophages (**B**) or of total T cells (% CD45^+^), FoxP3^+^ CD4^+^ T cells (%CD4^+^), and MFI of CD107a on CD45^+^ CD25^+^ CD8^+^ T cells (**C**) in the lung. Statistics: Two-way ANOVA followed by Tukey HSD. * *p* < 0.05; ** *p* < 0.01; *** *p* < 0.001; **** *p* < 0.0001.

Additionally, C/P, CDDO-Me, and the combination treatment markedly reduced PCNA expression in the tumors of WT mice (Figure 4A). A slight reduction in PCNA expression was also observed in Nrf2 KO mice treated with C/P, but not in Nrf2 KO mice treated with CDDO-Me alone. A small increase in TUNEL staining was observed in WT mice on combination treatment, but only a few cells per tumor were TUNELpositive (Figure S3). Phospho-ERK (p-ERK) staining was higher in WT mice treated with C/P. While CDDO-Me alone did not alter p-ERK expression, the combination of CDDO-Me and C/P decreased expression in lung sections from WT mice. No changes in p-ERK expression were observed in Nrf2 KO mice, regardless of treatment. In addition, there was a marked increase in p-ERK in Nrf2 KO mice in all treatment groups compared to WT mice (Figure 4A). These observations highlight the dependency on Nrf2 for the anti-tumor effects of CDDO-Me, and the increased p-ERK expression in Nrf2 KO mice showcases the enhanced Nrf2 activity in tumors with Ras activation.

### 3.4. CDDO-Me and Chemotherapy Alter Immune Cell Phenotype

To evaluate changes in the lung tumor immune microenvironment, lungs were analyzed by flow cytometry using an optimized antibody panel [30]. While the number of infiltrating (Figure 4B) and alveolar (Figure S4) macrophage numbers did not change, expression of the immunosuppressive marker PD-L1 and the tumor-promoting macrophage marker CD206 was significantly (*p* < 0.05) lower on infiltrating macrophages in the lungs of WT mice treated with CDDO-Me, C/P, or the combination (Figure 4B). Additionally, all 3 treatments decreased PD-L1 expression in alveolar macrophages in WT mice (Figure S4). No changes in PD-L1 or CD206 expression were observed in Nrf2 KO mice.

Excitingly, total T cell infiltration was higher in the lungs of WT mice treated with the combination of CDDO-Me and C/P (Figure 4C). Treatment with CDDO-Me, C/P, or the combination reduced the proportion of CD4+ T cells expressing the immunosuppressive regulatory T cell marker FoxP3. Not only was this change absent in the Nrf2 KO mice regardless of treatment, but a higher proportion of CD4+ T cells expressed FoxP3 in Nrf2 KO mice compared to WT mice (Figure 4C). Additionally, a greater proportion of activated CD8+ T cells expressed the degranulation marker CD107a, which correlates with an increased release of cytotoxic granules [37], in the lungs of WT mice treated with either C/P alone or the combination of CDDO-Me and C/P (Figure 4C and Figure S4). This change was absent in the Nrf2 KO mice, again indicating a dependency on Nrf2. Increased expression of CD107a was also observed in NK cells in the lungs of WT mice treated with C/P, CDDO-Me, and the combination (Figure S4). Interestingly, all treatments decreased the number of NK cells in the lungs of Nrf2 KO mice, and treatment with CDDO-Me reduced CD107a expression on NK cells present in the Nrf2 KO mice (Figure S4). These changes in various immune cell populations, coupled with the lack of difference in other immune cell populations including dendritic cells (Figure S5), showcase the complex network of immune cell communication that is affected by CDDO-Me treatment.

### 3.5. CDDO-Me Protects from Chemotherapy-Induced Toxicity and Mortality in WT but Not Nrf2 KO Mice

**Figure 5:**
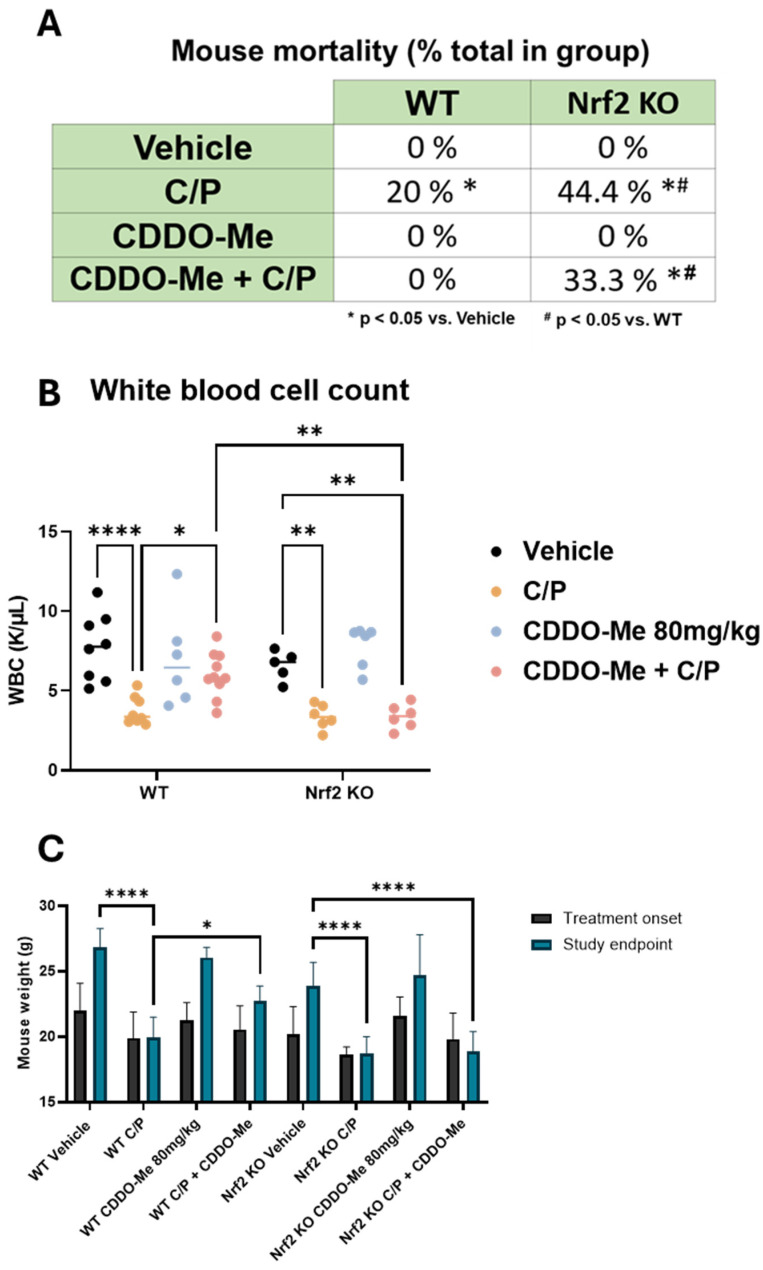
**CDDO-Me protects from chemotherapy-induced toxicity and mortality.** (**A**) Summary of mortality in wildtype (WT) and Nrf2 knockout (KO) A/J mice treated with CDDO-Me, carboplatin and paclitaxel (C/P), or the combination as described in Figure 3A; proportion of total enrolled in the treatment group at study initiation; n = 9–16 mice per group. (**B**) White blood cell (WBC) counts in whole blood measured at study endpoint. (**C**) Average mouse weights at beginning of treatment and at the study endpoint. Statistics: (**A**): z test for proportions. * *p* < 0.05 vs. vehicle; ^#^ *p* < 0.05 vs. WT. (**B**,**C**): Two-way ANOVA followed by Tukey HSD. * *p* < 0.05; ** *p* < 0.01; **** *p* < 0.0001.

A significant limitation to the efficacy of cytotoxic chemotherapy is the adverse effects caused by these drugs [38,39,40]. A/J mice are especially susceptible to many of these known toxicities. Indeed, adverse effects were observed in the mice treated with chemotherapy throughout the course of this study, and several animals either died or required euthanasia prior to the study endpoint in accordance with IACUC guidelines. Retrospective analysis of these events revealed three notable differences (Figure 5A). First, a significantly (*p* < 0.05) greater proportion of Nrf2 KO mice treated with C/P (44.4%) died throughout the study compared to WT mice treated with C/P (20%). Second, no WT mice treated with CDDO-Me or the combination of CDDO-Me plus C/P died, suggesting a protective effect of CDDO-Me. Third, there was no significant difference in the proportion of Nrf2 KO mice that died while receiving C/P alone (44.4%) compared to Nrf2 KO mice that were treated with the combination of CDDO-Me and C/P (33.3%). Taken together, these results suggest the protective effects against toxicity from carboplatin/paclitaxel chemotherapy observed in the WT mice are Nrf2-dependent.

Furthermore, complete blood counts using whole blood collected from animals immediately following euthanasia revealed a decreased white blood cell count in mice treated with C/P, regardless of genotype. However, combination treatment with CDDO-Me and C/P reversed this effect and rescued (*p* < 0.05) white blood cell counts in WT mice but not Nrf2 KO mice (Figure 5B). CDDO-Me alone did not change white blood cell count. Additionally, treatment with C/P decreased red blood cell counts, hemoglobin, and hematocrit and increased mean corpuscular volume/hemoglobin and red blood cell distribution width (Figure S6). In contrast to chemotherapy, where these changes are frequently observed [41,42,43], treatment with CDDO-Me alone did not change any of these blood count measures (Figure S6). Mirroring these trends, mouse weights were similarly adversely affected by C/P. Both WT and Nrf2 KO mice treated with C/P weighed significantly (*p* < 0.0001) less than mice on either vehicle or CDDO-Me intermittent diet at the end of the study. No significant difference was observed in the final weights of mice treated with CDDO-Me in the WT or Nrf2 KO mice vs. the respective vehicle control group. Moreover, the combination of CDDO-Me plus C/P partially rescued final mouse weights in WT but not Nrf2 KO mice (Figure 5C). These trends in mouse weight became apparent soon after the initiation of chemotherapy and endured throughout treatment (Figure S7).

### 3.6. Increased Tumor Burden and Histopathological Severity in Male vs. Female Mice

**Figure 6:**
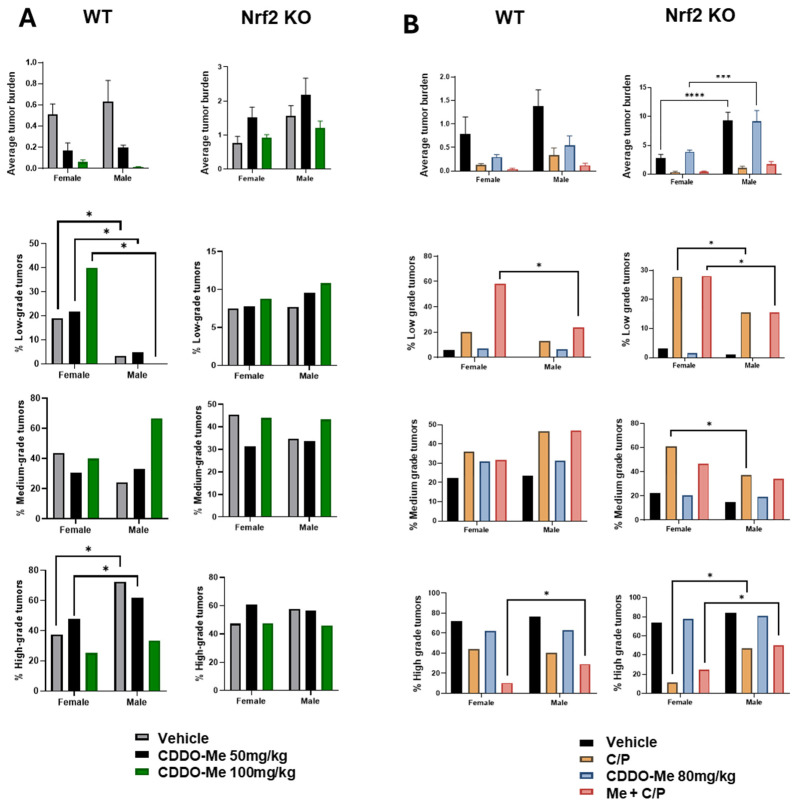
**Increased tumor burden and histopathological severity in male vs. female mice.** (**A**). Quantification of overall tumor burden and proportions of tumor histopathological grade in wildtype (WT) and Nrf2 knockout (KO) female and male mice treated with 50–100 mg/kg CDDO-Me for 8 weeks (**A**) or with 80 mg/kg ± C/P for 12 weeks (**B**). Statistics: Two-way ANOVA followed by Tukey HSD. *** *p* < 0.001; **** *p* < 0.0001. Z test for proportions. * *p* < 0.05.

Our previous studies detected a trend towards sex differences in the lung tumor burden of A/J mice challenged with vinyl carbamate [19], but these differences often did not reach statistical significance. Interestingly, sex differences observed in WT vs. Nrf2 KO mice in our current studies varied based on the length of treatment (8 vs. 12 weeks of treatment). After 8 weeks of treatment, statistically significant (*p* < 0.05) differences were observed in the histopathology of lung tumors in WT mice: a lower proportion of low-grade tumors in male vs. female WT mice, and the ratio of high-grade aggressive tumors was approximately 2-fold higher in males compared to females (Figure 6A, Tables S1 and S2). At the later endpoint of 12 weeks of treatment, male Nrf2 KO mice presented with a nearly two-fold increase (*p* < 0.0001) in overall tumor burden compared to female Nrf2 KO mice (Figure 6B, Table S3).

## 4. Discussion

The consequences of pharmacological NRF2 activation in cancer remains a controversial topic. Context plays an extremely important role in determining whether activation of the pathway has pro- or anti-tumor effects. Although NRF2 activation in cancer cells can promote drug resistance and tumor cell survival, NRF2 activation in the microenvironment can have the opposite effect [18,19,44,45,46,47]. As immune cells are particularly sensitive to the high levels of oxidative stress in the tumor microenvironment [11,12,13,48,49,50,51], NRF2 activation in immune cells can increase their tolerance of the harsh environment and facilitate an anti-tumor immune response.

With the transition of triterpenoids from bench to bedside with the FDA approval of omaveloxolone [52], understanding the biology of this class of drugs in the context of cancer is critical. CDDO-Me has been tested in clinical trials for many diseases, including cancer, pulmonary arterial hypertension, and chronic kidney disease. It is safe and well-tolerated in human patients, even at doses as high as 15 mg/d [53]. Although triterpenoids such as CDDO-Me are potent activators of the NRF2 pathway with well-documented anti-tumor activity in preclinical models of cancer [25,26,54,55,56,57], their effects on the immune microenvironment and the necessity of NRF2 for efficacy in treating established lung tumors were previously unknown.

Vinyl carbamate-challenged A/J mice are especially useful for studying lung carcinogenesis. Although urethane and the tobacco carcinogen 4-(methylnitrosamino)-1-(3-pyridyl)-1-butanone (NNK) [58] are frequently used to induce lung cancer in these genetically susceptible mice, they induce benign adenomas. In contrast, vinyl carbamate, an isocyanate found in cigarette smoke [58] and a metabolite of urethane [59], initiates the formation of aggressive adenocarcinomas in A/J mice, which increases the clinical relevance of the model as a majority of human lung cancers are adenocarcinomas. Additionally, vinyl carbamate induces a *Kras* mutation, which drives carcinogenesis in A/J mice; *KRAS* mutations represent a major subset of particularly aggressive human lung cancer cases [5]. The ability to evaluate *Kras*-driven disease progression and immune cell infiltration and phenotypes across different stages of lung cancer is uniquely advantageous as *Kras* mutations are known to alter the tumor immune microenvironment [60]. These characteristics make the A/J mouse model especially relevant for studying pharmacological Nrf2 activation in cancer. Additionally, the anti-tumor effects of CDDO-Me in A/J mice have been replicated in multiple studies [19,24,25].

In our previous early-stage tumorigenesis studies, low-dose (12.5–50 mg/kg of diet) CDDO-Me treatment was started three weeks post-initiation [19]. In our current studies, tumors developed over 10 weeks (two weeks of initiation plus eight weeks of tumor growth) before treatment. Because advanced tumors are harder to treat, higher doses of CDDO-Me were used than for previous prevention studies, and CDDO-Me was combined with C/P in study 2 in an attempt to regress established tumors. These differences in experimental design were critical, as NRF2 has distinct effects throughout carcinogenesis [21]. Our new results demonstrate that the anti-cancer efficacy of CDDO-Me is mostly dependent on Nrf2, even for the treatment of advanced tumors, and these results are consistent with our previous intervention studies with CDDO-Me in early-stage lung cancer.

CDDO-Me reduced tumor number, size, and burden in a dose-dependent manner in both studies (Figure 1 and Figure 3). The combination of CDDO-Me and C/P in study 2 was significantly more effective than either agent alone in WT mice, and the combination effect on tumor histopathological grade was striking (Table 2). Decreased proliferation, as determined by PCNA expression, in tumor cells (Figure 4A) but a lack of apoptosis (Figure S3) observed with CDDO-Me treatment alone suggested growth inhibition or tumor stasis but not tumor regression and thus warranted combination with the cytotoxic agents C/P. While C/P and CDDO-Me alone decreased PCNA expression, the combination further reduced proliferation in tumors and increased TUNEL staining. Importantly, the majority of the anti-tumor effects of CDDO-Me were lost in the Nrf2 KO mice in both studies, indicating that this mechanism is mostly dependent on Nrf2 (Table 1 and Table 2). Conversely, C/P was effective in both WT mice and Nrf2 KO mice in study 2, demonstrating an anti-tumor effect independent of Nrf2. Treatment with C/P alone increased p-ERK expression in WT tumors (Figure 4A), and paclitaxel is known to induce p-ERK [61]. However, there was a marked decrease in p-ERK expression in WT tumors of mice treated with the combination of CDDO-Me plus C/P. The distinction between the Nrf2-dependent mechanism with CDDO-Me and the Nrf2-independent mechanism with chemotherapy likely enhances the anti-tumor effect observed with the combination, as the different drug classes work independently to reduce tumor burden.

The slight decrease in the tumor burden of Nrf2 KO mice treated with CDDO-Me in study 2 (Table 2) was of particular interest. As the complete functional knockout of Nrf2 has been confirmed in these mice [19], these results suggest additional anti-tumor mechanisms of CDDO-Me independent of Nrf2. Interestingly, however, when combined with C/P, CDDO-Me did not enhance the reduction of tumor burden in Nrf2 KO mice. Different pharmacological Nrf2 activators can have different biological effects, likely a result of additional targets independent of Nrf2 [23,24,62,63,64]. Because the mechanism of triterpenoids such as CDDO-Me and many other natural products involves interaction with cysteine residues, a common mechanism of intracellular signal transduction, Nrf2 is not the lone target of this drug [23]. Transcriptomic sequencing is planned to help elucidate the effects of this drug on alternative targets. Interestingly, no Nrf2-independent effect of CDDO-Me was observed in study 1 even at the higher dose of 100 mg/kg, suggesting a temporal effect as the endpoint for study 1 was four weeks earlier than that of study 2. Notably, while slight reductions in tumor number and burden were observed in Nrf2 KO mice treated with CDDO-Me alone in study 2, no changes in immune cell profiles were observed in this group, suggesting that while CDDO-Me has secondary anti-tumor mechanisms, the immunomodulatory effects are dependent on Nrf2.

Distinct changes in immune cell populations were observed in this late-stage intervention model compared to the previously completed studies in the early-stage intervention, or prevention, model [19]. While the primary immunophenotypic changes were increased macrophage infiltration but decreased CD206 expression in the lungs of mice when treatment with CDDO-Me started 3 weeks after initiation [19], no change in macrophage infiltration was observed in our current late-stage intervention studies. Macrophage trafficking and activity promote early-stage lung tumorigenesis [65], but other immune cells such as T cells become more important in later stages. Tellingly, in this later-stage intervention model, a reduction in CD206 expression in macrophages was accompanied by a reduction in PD-L1 expression in the macrophages (Figure 2A and Figure 4B). CD206 is a marker of tumor-promoting macrophages which has been linked to worse patient prognosis in patients with solid cancers [33]. PD-L1 expression on tumor-infiltrating macrophages has suppressive effects on anti-tumor T cells within the lung tumor microenvironment and correlates with disease progression [35]. Reactive oxygen species (ROS), which increase in the tumor microenvironment as tumors progress, upregulate PD-L1 expression in macrophages [66], and Nrf2 activation in these cells can block the accumulation of ROS [67,68].

The reduction in PD-L1 expression on macrophages is consistent with an important role for T cells in established tumors. Veritably, the decrease in FoxP3-expressing CD4+ regulatory T cells coupled with increased expression of the degranulation marker CD107a on activated CD8+ T cells in WT mice treated with CDDO-Me and/or C/P (Figure 2B and Figure 4C) marked a favorable shift in T cell activity within the tumor microenvironment towards a more cytotoxic, anti-tumor phenotype. FoxP3+ regulatory T cells are immunosuppressive and correlate with worse patient prognosis [6,36], and the decrease in this population with CDDO-Me treatment favorably complements the increase in degranulation markers on cytotoxic T cells. Additionally, treatment with the combination of CDDO-Me plus C/P increased total T cell infiltration in the lungs of WT mice. In contrast, no changes in T cells, cell number, or phenotypic markers were observed in the early-stage intervention studies [19].

This time- and progression-dependent involvement of T cells is consistent with recent studies describing differing effects of Nrf2 activation in early- vs. late-phase T cell activation [69,70,71,72]. Clinical implications of these findings include the possibility of combining a triterpenoid with an immune checkpoint inhibitor in human lung cancer, as most human lung cancers are not diagnosed until they have reached late stages [1]. In study 2, increased expression of CD107a in NK cells in the lungs of mice treated with either CDDO-Me or the combination of CDDO-Me plus C/P (Figure S4) also illustrates the importance of temporally studying anti-tumor intervention with Nrf2 activators, as this change was only observed in WT mice with the longest treatment duration. Future directions include investigating the direct effects of CDDO-Me on NK cells, which are particularly sensitive to ROS in their environment. Overexposure to ROS without proper antioxidant mechanisms can lead to cell dysfunction and reduction in tumor-killing ability [54,73,74,75].

Our results suggest that the beneficial effects of pharmacological Nrf2 activation in the immune microenvironment may outweigh the survival advantage of Nrf2 activation for tumor cells, thereby slowing disease progression and leading to an overall reduction in tumor burden. In study 1, immunohistochemical staining of the Nrf2 target NQO1 in the lungs of WT mice illustrates that CDDO-Me (100 mg/kg) activated Nrf2 in both the tumor cells and the surrounding microenvironment (Figure 2A), yet the cytoprotective benefits of Nrf2 activation in the tumor were not sufficient to increase survival of these cells as tumor number, size, burden, and histopathological grade were significantly reduced in this treatment group (Figure 1 and Table 1).

The specific mechanism of how CDDO-Me alters phenotypes in macrophages and T cells within the lung tumor microenvironment, however, remains poorly understood. Our studies have shown that these effects on immune cells are Nrf2-dependent. In a non-cancer context, Nrf2 activation in macrophages and T cells has anti-inflammatory effects, as Nrf2 binds to DNA elements in the nucleus and blocks the translation of pro-inflammatory genes [76]. Additionally, an inverse relationship between the Nrf2 and NF-κB pathways mediates many pro-inflammatory pathways under the transcriptional control of NF-κB [77]. This regulation helps protect against the development of autoimmune disorders, and appropriate redox activity within immune cells, particularly T cells, is critical to prevent the development of systemic lupus erythematosus [78]. However, a highly oxidative and stressful environment such as that of the lung tumor microenvironment has suppressive effects on immune cells, as immune cells do not have a high capacity for tolerating oxidative stress [11,12,13,48,49,50,51]. Indeed, Nrf2 activation selectively in myeloid populations results in decreased tumors in the lung, demonstrating the complexity of this mechanism [18]. Additionally, selective activation of Nrf2 in FoxP3-expressing regulatory T cells results in enhanced effector T cell function and increased immune cell infiltrates in the lung [79]. We therefore hypothesize that the immune stimulatory effects observed in this model are not a direct result of Nrf2 binding response elements in the nucleus or directly modulating pro-inflammatory pathways, but rather a more complicated effect in which Nrf2 activation enables immune cells to better tolerate the stressful tumor microenvironment and therefore mount and maintain an appropriate anti-tumor response. Future studies are planned to test selective inhibition of Nrf2 in immune cells to answer these important questions.

Of additional interest was the detection of sex differences in tumor burden and histopathological grade in the mice (Figure 6). Interestingly, these changes were more apparent in WT or Nrf2 KO mice depending on study duration. While trends of higher tumor burden in male vs. female mice were found in both WT and Nrf2 KO mice, more statistically significant differences were found in WT mice in study 1. The endpoint of this study was only 8 weeks after the start of the treatment diet (a total of 18 weeks post-tumor initiation). Conversely, more significant differences in tumor burden and histopathological grade were observed in Nrf2 KO mice at the endpoint of study 2, after 12 weeks on the treatment diet (22 total weeks post-tumor initiation). In both WT and Nrf2 KO mice, male mice had a lower proportion of the least aggressive low-grade tumors and a higher proportion of high-grade tumors compared to female mice. These trends model epidemiological data in human lung cancer, in which males have a higher lifetime risk of developing lung cancer, present with more aggressive disease, and have a higher overall mortality compared to females [80,81,82]. However, little is known about how Nrf2 status affects tumorigenesis in males vs. females. Recent studies have suggested that males are more susceptible to oxidative stress [83,84,85,86], which could be exacerbated by more advanced lung cancer. Additionally, male mice in our studies were less responsive to chemotherapy compared to females. Although this trend did not reach statistical significance, it too follows human epidemiological data in which female patients tend to have a higher response rate to chemotherapy and better long-term clinical outcomes compared to males [87,88,89].

Regardless of sex, most lung cancer patients still receive chemotherapy, and carboplatin/paclitaxel remains one of the more commonly used cocktails [2]. While these drugs are effective at reducing tumor size, their systemic adverse effects are a significant limitation for their prolonged clinical use. Triterpenoids can help alleviate these adverse symptoms without having any detrimental effect on their anti-tumor efficacy [24,90]. Indeed, CDDO-Me protected mice from mortality, weight loss, and decreased white blood cell counts (Figure 5). The lack of protection in Nrf2 KO mice strongly suggests that the protective effects of CDDO-Me in chemotherapy-induced toxicities are dependent on Nrf2.

While the results of these studies are promising, certain limitations remain. Full-body Nrf2 KO does not allow investigation into the necessity of Nrf2 activation in specific cell types (e.g., tumor cells vs. immune cells) for the anti-tumor effects of CDDO-Me. Planned experiments with orthotopic models of Nrf2-active and Nrf2-null lung cancer cells will elucidate the effects of pharmacological Nrf2 activation in the microenvironment vs. genetic Nrf2 activation in tumor cells, a model that more accurately reflects human lung cancers with Nrf2-activating mutations. Additionally, while no detectable resistance to treatment (neither CDDO-Me nor C/P) was observed, chemoresistance remains a significant challenge in clinical cancer management. A/J mice are not suitable for studying chemoresistance, as these tumors are slow-growing and difficult to evaluate longitudinally. Therefore, we will utilize a faster-growing orthotopic lung model to elucidate these mechanisms.

The results of these studies suggest testing CDDO-Me in combination with other anti-cancer therapies currently used to treat patients with lung cancer, particularly cytotoxic chemotherapy and immune checkpoint blockade. The value of including CDDO-Me in combination treatment regimens includes reduction of toxicity and enhanced anti-tumor activity through the addition of different pharmacological mechanisms of action. Clinical trials have demonstrated that CDDO-Me is safe in human patients at high doses both during treatment and after long-term follow-up. However, the differences between murine and human biology in disease pathology and response to different treatment combinations resulting from species differences including pharmacokinetics, treatment duration, and overall tumor size remain a challenge. The increased diversity in human lung cancers compared to murine models requires careful consideration of which patient populations would benefit from treatment with CDDO-Me or a related triterpenoid.

## 5. Conclusions

The current studies suggest that pharmacological Nrf2 activation in an advanced lung cancer model decreases tumor burden through several mechanisms including favorable immunomodulation in the tumor microenvironment, reduced tumor cell proliferation, and reduced activity of the RAS pathway. While most studies evaluating Nrf2 in cancer focus on genetic Nrf2 activation in tumor cells, we used pharmacological Nrf2 activation and studied effects in the tumor microenvironment. Importantly, CDDO-Me adds to the anti-tumor efficacy of conventional cytotoxic chemotherapy drugs while simultaneously protecting mice from toxicity. The combination effects of CDDO-Me with chemotherapy demonstrate the potential advantages of combining triterpenoids with other anti-cancer treatments. Particularly, the immunomodulatory effects of CDDO-Me on T cells suggest that combining a triterpenoid with an immune checkpoint inhibitor may be even more effective for treating advanced lung cancer. Additionally, the observed p-ERK inhibition suggests that the combination of CDDO-Me with a KRAS inhibitor may have complementary effects [91]. Future studies are planned to test these hypotheses.

## Figures and Tables

**Table 1:** **CDDO-Me reduces lung tumor burden and severity of the tumor histopathology in a dose- and Nrf2-dependent manner.** Wildtype (WT) and Nrf2 knockout (KO) A/J mice were challenged with vinyl carbamate to induce lung carcinogenesis as shown in Figure 1. After two separate cohorts of WT and Nrf2 KO mice with established lung tumors were fed either a control diet or diet containing (**A**) 50 mg/kg CDDO-Me or (**B**) 100 mg/kg CDDO-Me for 8 weeks, tumor number on both the surface of the lungs and on tumor sections as well as tumor size, overall tumor burden, and histopathological grades on tumor sections were quantified.

A	WT Control	WT CDDO-Me 50 mg/kg	Nrf2 KO Control	Nrf2 KO CDDO-Me 50 mg/kg
Total surface tumors	352	231	812	856
Average # of tumors per mouse (% WT Control)	25.14 ± 1.3 (100%)	16.5 ± 1.3 (65.6%) *	62.46 ± 2.6 (248.4%) ^####^	61.14 ± 3.2 (243.2%) ^####^
# of mice per group	14	14	13	14
Average # tumors/slide (% WT Control)	2.18 ± 0.2 (100%)	1.57 ± 0.3 (72.1%)	5.04 ± 0.2 (231.3%) ^####^	5.54 ± 0.2 (254.1%) ^####^
Average Tumor Size (mm^3^) (% WT Control)	0.26 ± 0.08 (100%)	0.12 ± 0.05 (45.2%)	0.35 ± 0.1 (133.1%)	0.33 ± 0.1 (127.6%) ^#^
Average Tumor Burden (mm^3^) (% WT Control)	0.57 ± 0.3 (100%)	0.19 ± 0.3 (32.6%)	1.75 ± 0.5 (307.7%) ^#^	1.85 ± 0.6 (324.11%) ^#^
Low Grade (% total)	11.5	13.6	7.6	9.0
Medium Grade (% total)	34.4	31.8	38.9	32.9
High Grade (% total)	54.1	54.6	53.5	58.1
**B**	**WT Control**	**WT CDDO-Me 100 mg/kg**	**Nrf2 KO Control**	**Nrf2 KO CDDO-Me 100 mg/kg**
Total surface tumors	403	125	799	650
Average # of tumors per mouse (% WT Control)	22.4 ± 1.3 (100%)	6.9 ± 1.0 (31%) ****	53.3 ± 2.6 (237.9%) ^####^	54.17 ± 2.4 (241.9%) ^####^
# of mice per group	18	18	15	12
Average # tumors/slide (% WT Control)	2.3 ± 0.3 (100%)	0.64 ± 0.2 (27.4%) ***	4.07 ± 0.5 (174.3%) ^###^	3.92 ± 0.4 (167.9%) ^####^
Average Tumor Size (mm^3^) (% WT Control)	0.17 ± 0.09 (100%)	0.07 ± 0.02 (39.7%) *	0.29 ± 0.02 (170.7%)	0.26 ± 0.1 (151.47%) ^###^
Average Tumor Burden (mm^3^) (% WT Control)	0.4 ± 0.09 (100%)	0.04 ± 0.02 (10.9%) ***	1.19 ± 0.2 (297.5%) ^####^	1.01 ± 0.1 (151.5%) ^####^
Low Grade (% total)	14.3	34.8 *	9.7	9.6 ^#^
Medium Grade (% total)	46.4	39.1	42.3	43.6
High Grade (% total)	39.3	26.1 *	48.0	46.8 ^#^

**Table 1 statistics**: Two-way ANOVA followed by Tukey HSD (tumor number, size, and burden): * *p* < 0.05 vs. WT Control; *** *p* < 0.001 vs. WT Control; **** *p* < 0.0001 vs. WT Control. ^#^ *p* < 0.05 Nrf2 KO vs. WT; ^###^ *p* < 0.001 Nrf2 KO vs. WT; ^####^ *p* < 0.0001 Nrf2 KO vs. WT. Z test for proportions (tumor histopathological grades): * *p* < 0.05 vs. WT Vehicle; ^#^ *p* < 0.05 Nrf2 KO vs. WT.

**Table 2:** **CDDO-Me, alone and in combination with carboplatin and paclitaxel (C/P), reduces lung tumor burden and severity of tumor histopathology**. Wildtype (WT) and Nrf2 knockout (KO) A/J mice were challenged with vinyl carbamate to induce lung carcinogenesis as shown in Figure 3. Quantification of tumor number, tumor size, overall tumor burden, and tumor histopathological grades.

	WT Control	WT C/P	WT CDDO-Me 80 mg/kg	WT CDDO-Me + C/P	Nrf2 KO Control	Nrf2 KO C/P	Nrf2 KO CDDO-Me 80 mg/kg	Nrf2 KO CDDO-Me + C/P
Total surface tumors	639	227	231	75	826	271	664	296
Average per mouse (% WT Control)	39.9 ± 2.0 (100%)	18.9 ± 1.9 (47.4) ****	21 ± 1.2 (52.6%) ****	5.8 ± 1.1 (14.5%) **** ^!^	91.8 ± 4.2 (229.8%) ^####^	38.7 ± 2.6 (96.9%) ^# $$$$^	73.8 ± 6.2 (184.7%)	37 ± 3.9 (92.6%) ^#### $$$$^
# of mice per group	16	12	10	13	9	7	9	8
Average # of tumors per slide (% WT Control)	2.59 ± 0.28 (100%)	1.67 ± 0.17 (64.3%) *	2.25 ± 0.32 (86.8%)	1.38 ± 0.24 (53.38%) *	7.39 ± 0.78 (284.9%) ^####^	3.57 ± 0.64 (137.7%) ^### $$$$^	6.67 ± 0.51 (257%) ^####^	4.00 ± 0.54 (154.2%) ^#### $$$$^
Average Tumor Size (mm^3^/tumor) (% WT Control)	0.42 ± 0.16 (100%)	0.12 ± 0.04 (29.1%) **	0.17 ± 0.05 (39.7%) *	0.05 ± 0.02 (12.4%) ****	0.97 ± 0.45 (232%) ^##^	0.19 ± 0.09 (44.9%) * ^$$$$^	0.85 ± 0.33 (203.8%) ^###^	0.23 ± 0.12 (55.8%) ^# $$$$^
Average Tumor Burden (mm^3^) (% WT Control)	1.09 ± 0.25 (100%)	0.20 ± 0.06 (18.7%) ****	0.37 ± 0.07 (34.4%) ***	0.07 ± 0.02 (6.6%) **** ^!^	7.17 ± 1.2 (660.9%) ^####^	0.67 ± 0.18 (61.8%) ^# $$$$^	5.68 ± 0.86 (523.8%) ^$ ####^	0.93 ± 0.24 (86%) ^####^
Low Grade (% total)	2.4	17.5 *	6.7	41.7 * ^!^	1.5	20 ^$^	0.8^#^	21.9^# $^
Medium Grade (% total)	22.9	40 *	31.1 *	38.9 *	16.5	46 ^$^	20.0	40.6 ^$^
High Grade (% total)	74.7	42.5 *	62.2 *	19.4 * ^!^	82	34 ^$^	79.2	37.5 ^# $^

**Table 2 statistics:** Two-way ANOVA followed by Tukey HSD (tumor number, size, and burden): * *p* < 0.05 vs. WT Vehicle; ** *p* < 0.01 vs. WT Vehicle; *** *p* < 0.001 vs. WT Vehicle; **** *p* < 0.0001 vs. WT Vehicle; ^#^ *p* < 0.05 Nrf2 KO vs. WT; ^##^ *p* < 0.01 Nrf2 KO vs. WT; ^###^ *p* < 0.001 Nrf2 KO vs. WT; ^####^ *p* < 0.0001 Nrf2 KO vs. WT; ^!^ *p* < 0.05 vs. WT C/*p* and WT CDDO-Me 80 mg/kg; ^$$$$^ *p* < 0.0001 vs. Nrf2 KO Control. Z test for proportions (tumor histopathological grades): * *p* < 0.05 vs. WT Vehicle; ^!^ *p* < 0.05 vs. WT C/P and WT CDDO-Me 80 mg/kg; ^#^ *p* < 0.05 Nrf2 KO vs. WT; ^$^ *p* < 0.05 vs. Nrf2 KO Control.

## Data Availability

Any relevant data not presented in this study are available by request from the corresponding author.

## Supplementary Materials

The following supporting information can be downloaded at: https://www.mdpi.com/article/10.3390/antiox13060621/s1, Figure S1: Weights of wildtype (WT) and Nrf2 knockout (KO) mice treated with vehicle or 50–100 mg/kg CDDO-Me for 8 weeks; Figure S2: Histopathological grades of lung tumors in WT and Nrf2 KO mice treated with vehicle and 50 mg/kg CDDO-Me for 8 weeks; Figure S3: Immunofluorescent staining for TUNEL in WT mice treated with vehicle, 80 mg/kg CDDO-Me, carboplatin and paclitaxel, or the combination for 12 weeks; Figure S4: Flow cytometric analysis of alveolar macrophages, CD8+ T cells, and NK cells in WT and Nrf2 KO mice treated with vehicle, 80 mg/kg CDDO-Me, carboplatin and paclitaxel, or the combination for 12 weeks; Figure S5: Dendritic cells in the lungs of mice; Figure S6: Complete blood counts in WT and Nrf2 KO mice treated with vehicle, 80 mg/kg CDDO-Me, carboplatin and paclitaxel, or the combination for 12 weeks; Figure S7: Weights measured once per week throughout the duration of the study of WT and Nrf2 KO mice treated with vehicle, 80 mg/kg CDDO-Me, carboplatin and paclitaxel, or the combination for 12 weeks; Table S1: Number, size, and histopathology of lung tumors in female and male WT and Nrf2 KO mice treated with vehicle and 50 mg/kg CDDO-Me for 8 weeks; Table S2: Number, size, and histopathology of lung tumors in female and male WT and Nrf2 KO mice treated with vehicle and 100 mg/kg CDDO-Me for 8 weeks; Table S3: Number, size, and histopathology of lung tumors in female and male WT and Nrf2 KO mice treated with vehicle, 80 mg/kg CDDO-Me, carboplatin and paclitaxel, or the combination for 12 weeks.

## References

[1] Siegel R.L., Giaquinto A.N., Jemal A. (2024). Cancer Statistics, 2024. CA Cancer J. Clin..

[2] Nawaz K., Webster R.M. (2023). The Non-Small-Cell Lung Cancer Drug Market. Nat. Rev. Drug Discov..

[3] Casaluce F., Gridelli C. (2023). Combined Chemo-Immunotherapy in Advanced Non-Small Cell Lung Cancer: Feasible in the Elderly?. Expert. Opin. Emerg. Drugs.

[4] Wang X., Qiao Z., Aramini B., Lin D., Li X., Fan J. (2023). Potential Biomarkers for Immunotherapy in Non-Small-Cell Lung Cancer. Cancer Metastasis Rev..

[5] Di Federico A., Ricciotti I., Favorito V., Michelina S.V., Scaparone P., Metro G., De Giglio A., Pecci F., Lamberti G., Ambrogio C. (2023). Resistance to Kras G12c Inhibition in Non-Small Cell Lung Cancer. Curr. Oncol. Rep..

[6] Altorki N.K., Markowitz G.J., Gao D., Port J.L., Saxena A., Stiles B., McGraw T., Mittal V. (2019). The Lung Microenvironment: An Important Regulator of Tumour Growth and Metastasis. Nat. Rev. Cancer.

[7] Shinohara S., Takahashi Y., Komuro H., Matsui T., Sugita Y., Demachi-Okamura A., Muraoka D., Takahara H., Nakada T., Sakakura N. (2022). New Evaluation of the Tumor Immune Microenvironment of Non-Small Cell Lung Cancer and Its Association with Prognosis. J. ImmunoTherapy Cancer.

[8] Wu Y., Yuan M., Wang C., Chen Y., Zhang Y., Zhang J. (2023). T Lymphocyte Cell: A Pivotal Player in Lung Cancer. Front. Immunol..

[9] Essogmo F.E., Zhilenkova A.V., Tchawe Y.S.N., Owoicho A.M., Rusanov A.S., Boroda A., Pirogova Y.N., Sangadzhieva Z.D., Sanikovich V.D., Bagmet N.N. (2023). Cytokine Profile in Lung Cancer Patients: Anti-Tumor and Oncogenic Cytokines. Cancers.

[10] Cui Z., Ruan Z., Li M., Ren R., Ma Y., Zeng J., Sun J., Ye W., Xu W., Guo X. (2023). Intermittent Hypoxia Inhibits Anti-Tumor Immune Response Via Regulating Pd-L1 Expression in Lung Cancer Cells and Tumor-Associated Macrophages. Int. Immunopharmacol..

[11] Yang Y., Bazhin A.V., Werner J., Karakhanova S. (2013). Reactive Oxygen Species in the Immune System. Int. Rev. Immunol..

[12] Yarosz E.L., Chang C.-H. (2018). The Role of Reactive Oxygen Species in Regulating T Cell-Mediated Immunity and Disease. Immune Netw..

[13] Wang L., Kuang Z., Zhang D., Gao Y., Ying M., Wang T. (2021). Reactive Oxygen Species in Immune Cells: A New Antitumor Target. Biomed. Pharmacother..

[14] Suzuki T., Takahashi J., Yamamoto M. (2023). Molecular Basis of the Keap1-Nrf2 Signaling Pathway. Mol. Cells.

[15] Adinolfi S., Patinen T., Deen A.J., Pitkänen S., Härkönen J., Kansanen E., Küblbeck J., Levonen A.L. (2023). The Keap1-Nrf2 Pathway: Targets for Therapy and Role in Cancer. Redox Biol..

[16] Sánchez-Ortega M., Carrera A.C., Garrido A. (2021). Role of Nrf2 in Lung Cancer. Cells.

[17] Brüne B., Dehne N., Grossmann N., Jung M., Namgaladze D., Schmid T., von Knethen A., Weigert A. (2013). Redox Control of Inflammation in Macrophages. Antioxid. Redox Signal.

[18] Hayashi M., Kuga A., Suzuki M., Panda H., Kitamura H., Motohashi H., Yamamoto M. (2020). Microenvironmental Activation of Nrf2 Restricts the Progression of Nrf2-Activated Malignant Tumors. Cancer Res..

[19] Moerland J.A., Leal A.S., Lockwood B., Demireva E.Y., Xie H., Krieger-Burke T., Liby K.T. (2023). The Triterpenoid Cddo-Methyl Ester Redirects Macrophage Polarization and Reduces Lung Tumor Burden in a Nrf2-Dependent Manner. Antioxidants.

[20] Liu L., Chen G., Gong S., Huang R., Fan C. (2023). Targeting Tumor-Associated Macrophage: An Adjuvant Strategy for Lung Cancer Therapy. Front. Immunol..

[21] Occhiuto C.J., Moerland J.A., Leal A.S., Gallo K.A., Liby K.T. (2023). The Multi-Faceted Consequences of Nrf2 Activation Throughout Carcinogenesis. Mol. Cells.

[22] Robertson H., Dinkova-Kostova A.T., Hayes J.D. (2020). Nrf2 and the Ambiguous Consequences of Its Activation During Initiation and the Subsequent Stages of Tumourigenesis. Cancers.

[23] Liby K.T., Sporn M.B. (2012). Synthetic Oleanane Triterpenoids: Multifunctional Drugs with a Broad Range of Applications for Prevention and Treatment of Chronic Disease. Pharmacol. Rev..

[24] Liby K.T. (2014). Synthetic Triterpenoids Can Protect against Toxicity without Reducing the Efficacy of Treatment with Carboplatin and Paclitaxel in Experimental Lung Cancer. Dose Response.

[25] Liby K., Royce D.B., Williams C.R., Risingsong R., Yore M.M., Honda T., Gribble G.W., Dmitrovsky E., Sporn T.A., Sporn M.B. (2007). The Synthetic Triterpenoids CDDO-Methyl Ester and CDDO-Ethyl Amide Prevent Lung Cancer Induced by Vinyl Carbamate in A/J Mice. Cancer Res..

[26] Plehn S., Wagle S., Rupasinghe H.P.V. (2023). Chaga Mushroom Triterpenoids as Adjuncts to Minimally Invasive Cancer Therapies: A Review. Curr. Res. Toxicol..

[27] Altun İ., Sonkaya A. (2018). The Most Common Side Effects Experienced by Patients Were Receiving First Cycle of Chemotherapy. Iran. J. Public Health.

[28] Jiang S., Pan A.W., Lin T.Y., Zhang H., Malfatti M., Turteltaub K., Henderson P.T., Pan C.X. (2015). Paclitaxel Enhances Carboplatin-DNA Adduct Formation and Cytotoxicity. Chem. Res. Toxicol..

[29] Titis A.P., Forkert P.-G. (2001). Strain-Related Differences in Bioactivation of Vinyl Carbamate and Formation of DNA Adducts in Lungs of a/J, Cd-1, and C57bl/6 Mice. Toxicol. Sci..

[30] Yu Y.R., O’Koren E.G., Hotten D.F., Kan M.J., Kopin D., Nelson E.R., Que L., Gunn M.D. (2016). A Protocol for the Comprehensive Flow Cytometric Analysis of Immune Cells in Normal and Inflamed Murine Non-Lymphoid Tissues. PLoS ONE.

[31] Taguchi K., Motohashi H., Yamamoto M. (2011). Molecular mechanisms of the Keap1–Nrf2 pathway in stress response and cancer evolution. Genes Cells.

[32] Jaiswal A.K. (2000). Regulation of genes encoding NAD(P)H:quinone oxidoreductases. Free Rad. Bio Med..

[33] Debacker J.M., Gondry O., Lahoutte T., Keyaerts M., Huvenne W. (2021). The Prognostic Value of CD206 in Solid Malignancies: A Systematic Review and Meta-Analysis. Cancers.

[34] Diskin B., Adam S., Cassini M.F., Sanchez G., Liria M., Aykut B., Buttar C., Li E., Sundberg B., Salas R.D. (2020). Pd-L1 Engagement on T Cells Promotes Self-Tolerance and Suppression of Neighboring Macrophages and Effector T Cells in Cancer. Nat. Immunol..

[35] Shinchi Y., Ishizuka S., Komohara Y., Matsubara E., Mito R., Pan C., Yoshii D., Yonemitsu K., Fujiwara Y., Ikeda K. (2022). The expression of PD-1 ligand 1 on macrophages and its clinical impacts and mechanisms in lung adenocarcinoma. Cancer Immunol. Immunother..

[36] Sarkar T., Dhar S., Sa G. (2021). Tumor-Infiltrating T-Regulatory Cells Adapt to Altered Metabolism to Promote Tumor-Immune Escape. Curr. Res. Immunol..

[37] Aktas E., Kucuksezer U.C., Bilgic S., Erten G., Deniz G. (2009). Relationship between Cd107a Expression and Cytotoxic Activity. Cell. Immunol..

[38] Zubair H.M., Khan M.A., Gulzar F., Alkholief M., Malik A., Akhtar S., Sharif A., Akhtar M.F., Abbas M. (2023). Patient Perspectives and Side-Effects Experience on Chemotherapy of Non-Small Cell Lung Cancer: A Qualitative Study. Cancer Manag. Res..

[39] Sin C., Kim H., Im H.S., Ock M., Koh S.J. (2023). Development and Pilot Study of “Smart Cancer Care”: A Platform for Managing Side Effects of Chemotherapy. BMC Health Serv. Res..

[40] Jachowski A., Marcinkowski M., Szydłowski J., Grabarczyk O., Nogaj Z., Marcin Ł., Pławski A., Jagodziński P.P., Słowikowski B.K. (2023). Modern Therapies of Nonsmall Cell Lung Cancer. J. Appl. Genet..

[41] Skverchinskaya E., Levdarovich N., Ivanov A., Mindukshev I., Bukatin A. (2023). Anticancer Drugs Paclitaxel, Carboplatin, Doxorubicin, and Cyclophosphamide Alter the Biophysical Characteristics of Red Blood Cells, in Vitro. Biology.

[42] Pirker R., Pirolli M., Quigley J., Hulnick S., Legg J., Collins H., Vansteenkiste J. (2013). Hemoglobin Decline in Cancer Patients Receiving Chemotherapy without an Erythropoiesis-Stimulating Agent. Support. Care Cancer.

[43] Groopman J.E., Itri L.M. (1999). Chemotherapy-Induced Anemia in Adults: Incidence and Treatment. JNCI J. Natl. Cancer Inst..

[44] Härkönen J., Pölönen P., Deen A.J., Selvarajan I., Teppo H.R., Dimova E.Y., Kietzmann T., Ahtiainen M., Väyrynen J.P., Väyrynen S.A. (2023). A Pan-Cancer Analysis Shows Immunoevasive Characteristics in Nrf2 Hyperactive Squamous Malignancies. Redox Biol..

[45] Baird L., Taguchi K., Zhang A., Takahashi Y., Suzuki T., Kensler T.W., Yamamoto M. (2023). A Nrf2-Induced Secretory Phenotype Activates Immune Surveillance to Remove Irreparably Damaged Cells. Redox Biol..

[46] Baird L., Yamamoto M. (2023). Immunoediting of Keap1-Nrf2 Mutant Tumours Is Required to Circumvent Nrf2-Mediated Immune Surveillance. Redox Biol..

[47] Lin L., Wu Q., Lu F., Lei J., Zhou Y., Liu Y., Zhu N., Yu Y., Ning Z., She T. (2023). Nrf2 Signaling Pathway: Current Status and Potential Therapeutic Targetable Role in Human Cancers. Front. Oncol..

[48] Morris G., Gevezova M., Sarafian V., Maes M. (2022). Redox Regulation of the Immune Response. Cell. Mol. Immunol..

[49] Nakamura K., Matsunaga K. (1998). Susceptibility of Natural Killer Cells to Reactive Oxygen Species and Their Restoration by the Mimics of Superoxide Dismutase. Cancer Biother. Radiopharm..

[50] Jiang S. (2020). Mitochondrial Oxidative Phosphorylation Is Linked to T-Cell Exhaustion. Aging.

[51] Kesarwani P., Murali A.K., Al-Khami A.A., Mehrotra S. (2013). Redox Regulation of T-Cell Function: From Molecular Mechanisms to Significance in Human Health and Disease. Antioxid. Redox Signal.

[52] Subramony S.H., Lynch D.L. (2023). A Milestone in the Treatment of Ataxias: Approval of Omaveloxolone for Friedreich Ataxia. Cerebellum.

[53] Nangaku M., Kanda H., Takama H., Ichikawa T., Hase H., Akizawa T. (2020). Randomized Clinical Trial on the Effect of Bardoxolone Methyl on GFR in Diabetic Kidney Disease Patients (TSUBAKI Study). Kidney Int. Rep..

[54] Nagaraj S., Youn J.I., Weber H., Iclozan C., Lu L., Cotter M.J., Meyer C., Becerra C.R., Fishman M., Antonia S. (2010). Anti-Inflammatory Triterpenoid Blocks Immune Suppressive Function of MDSCs and Improves Immune Response in Cancer. Clin. Cancer Res..

[55] Ball M.S., Bhandari R., Torres G.M., Martyanov V., ElTanbouly M.A., Archambault K., Whitfield M.L., Liby K.T., Pioli P.A. (2020). Cddo-Me Alters the Tumor Microenvironment in Estrogen Receptor Negative Breast Cancer. Sci. Rep..

[56] Wang Y.-Y., Zhe H., Zhao R. (2014). Preclinical Evidences toward the Use of Triterpenoid CDDO-Me for Solid Cancer Prevention and Treatment. Mol. Cancer.

[57] Bishayee A., Ahmed S., Brankov N., Perloff M. (2011). Triterpenoids as Potential Agents for the Chemoprevention and Therapy of Breast Cancer. Front. Biosci..

[58] Politi K., Cruz C.S.D., Homer R., McManus L.M., Mitchell R.N. (2014). Thoracic Neoplasia: Carcinoma. Pathobiology of Human Disease.

[59] Dahl G.A., Miller J.A., Miller E.C. (1978). Vinyl Carbamate as a Promutagen and a More Carcinogenic Analog of Ethyl Carbamate. Cancer Res..

[60] Hamarsheh S., Groß O., Brummer T., Zeiser R. (2020). Immune Modulatory Effects of Oncogenic Kras in Cancer. Nat. Commun..

[61] Okano J., Rustgi A.K. (2001). Paclitaxel Induces Prolonged Activation of the Ras/Mek/Erk Pathway Independently of Activating the Programmed Cell Death Machinery. J. Biol. Chem..

[62] To C., Ringelberg C.S., Royce D.B., Williams C.R., Risingsong R., Sporn M.B., Liby K.T. (2015). Dimethyl Fumarate and the Oleanane Triterpenoids, CDDO-Imidazolide and CDDO-Methyl Ester, Both Activate the Nrf2 Pathway but Have Opposite Effects in the A/J Model of Lung Carcinogenesis. Carcinogenesis.

[63] Pouremamali F., Pouremamali A., Dadashpour M., Soozangar N., Jeddi F. (2022). An Update of Nrf2 Activators and Inhibitors in Cancer Prevention/Promotion. Cell Commun. Signal..

[64] Dinkova-Kostova A.T., Copple I.M. (2023). Advances and Challenges in Therapeutic Targeting of Nrf2. Trends Pharmacol. Sci..

[65] Sedighzadeh S.S., Khoshbin A.P., Razi S., Keshavarz-Fathi M., Rezaei N. (2021). A Narrative Review of Tumor-Associated Macrophages in Lung Cancer: Regulation of Macrophage Polarization and Therapeutic Implications. Transl. Lung Cancer Res..

[66] Roux C., Jafari S.M., Shinde R., Duncan G., Cescon D.W., Silvester J., Chu M.F., Hodgson K., Berger T., Wakeham A. (2019). Reactive Oxygen Species Modulate Macrophage Immunosuppressive Phenotype through the up-Regulation of Pd-L1. Proc. Natl. Acad. Sci. USA.

[67] Canton M., Sánchez-Rodríguez R., Spera I., Venegas F.C., Favia M., Viola A., Castegna A. (2021). Reactive Oxygen Species in Macrophages: Sources and Targets. Front. Immunol..

[68] Wang P., Geng J., Gao J., Zhao H., Li J., Shi Y., Yang B., Xiao C., Linghu Y., Sun X. (2019). Macrophage Achieves Self-Protection against Oxidative Stress-Induced Ageing through the Mst-Nrf2 Axis. Nat. Commun..

[69] Gnanaprakasam J.N.R., Kushwaha B., Liu L., Chen X., Kang S., Wang T., Cassel T.A., Adams C.M., Higashi R.M., Scott D.A. (2023). Asparagine Restriction Enhances Cd8^+^ T Cell Metabolic Fitness and Antitumoral Functionality through an Nrf2-Dependent Stress Response. Nat. Metab..

[70] Morzadec C., Macoch M., Sparfel L., Kerdine-Römer S., Fardel O., Vernhet L. (2014). Nrf2 Expression and Activity in Human T Lymphocytes: Stimulation by T Cell Receptor Activation and Priming by Inorganic Arsenic and Tert-Butylhydroquinone. Free Radic. Biol. Med..

[71] Turley A.E., Zagorski J., Rockwell C.E. (2017). The Role of Nrf2 in Primary Human Cd4 T Cell Activation and Differentiation. FASEB J..

[72] Pant A., Dasgupta D., Tripathi A., Pyaram K. (2023). Beyond Antioxidation: Keap1-Nrf2 in the Development and Effector Functions of Adaptive Immune Cells. Immunohorizons.

[73] Whiteside T.L. (2020). Nk Cells in the Tumor Microenvironment and Thioredoxin Activity. J. Clin. Investig..

[74] Lu Z., Tian Y., Bai Z., Liu J., Zhang Y., Qi J., Jin M., Zhu J., Li X. (2022). Increased Oxidative Stress Contributes to Impaired Peripheral Cd56(Dim)Cd57(+) Nk Cells from Patients with Systemic Lupus Erythematosus. Arthritis Res. Ther..

[75] Stefanie R., Takahiro N., Isabelle M., Jonas M., Andreas L., Elias S.J.A., Rolf K., Linnea W.S. (2022). Targeting of Nrf2 Improves Antitumoral Responses by Human Nk Cells, Til and Car T Cells During Oxidative Stress. J. Immunother. Cancer.

[76] Kobayashi E., Suzuki T., Funayama R., Nagashima T., Hayashi M., Sekine H., Tanaka N., Moriguchi T., Motohashi H., Nakayama K. (2016). Nrf2 suppresses macrophage inflammatory response by blocking proinflammatory cytokine transcription. Nat. Commun..

[77] Wardyn J.D., Ponsford A.H., Sanderson C.M. (2015). Dissecting molecular cross-talk between Nrf2 and NF-κB response pathways. Biochem. Soc. Trans..

[78] Yoh K., Itoh K., Enomoto A., Hirayama A., Yamaguchi N., Kobayashi M., Morito N., Koyama A., Yamamoto M., Takahashi S. (2001). Nrf2-deficient female mice develop lupus-like autoimmune nephritis. Kidney Int..

[79] Klemm P., Rajendiran A., Fragoulis A., Wruck C., Schippers A., Wagner N., Bopp T., Tenbrock K., Ohl K. (2020). Nrf2 expression driven by Foxp3 specific deletion of Keap1 results in loss of immune tolerance in mice. Eur. J. Immunol..

[80] May L., Shows K., Nana-Sinkam P., Li H., Landry J.W. (2023). Sex Differences in Lung Cancer. Cancers.

[81] Poleri C. (2022). Sex-Based Differences in Lung Cancer: Does It Matter?. J. Thorac. Oncol..

[82] Stabellini N., Bruno D.S., Dmukauskas M., Barda A.J., Cao L., Shanahan J., Waite K., Montero A.J., Barnholtz-Sloan J.S. (2022). Sex Differences in Lung Cancer Treatment and Outcomes at a Large Hybrid Academic-Community Practice. JTO Clin. Res. Rep..

[83] Allegra A., Caserta S., Genovese S., Pioggia G., Gangemi S. (2023). Gender Differences in Oxidative Stress in Relation to Cancer Susceptibility and Survival. Antioxidants.

[84] Ali I., Högberg J., Hsieh J.-H., Auerbach S., Korhonen A., Stenius U., Silins I. (2016). Gender Differences in Cancer Susceptibility: Role of Oxidative Stress. Carcinogenesis.

[85] Martínez de Toda I., González-Sánchez M., Cerro E.D.-D., Valera G., Carracedo J., Guerra-Pérez N. (2023). Sex Differences in Markers of Oxidation and Inflammation. Implications for Ageing. Mech. Ageing Dev..

[86] Zabłocka-Słowińska K., Płaczkowska S., Skórska K., Prescha A., Pawełczyk K., Porębska I., Kosacka M., Grajeta H. (2019). Oxidative Stress in Lung Cancer Patients Is Associated with Altered Serum Markers of Lipid Metabolism. PLoS ONE.

[87] Rakshith H.T., Lohita S., Rebello A.P., Goudanavar P.S., Naveen N.R. (2023). Sex Differences in Drug Effects and/or Toxicity in Oncology. Curr. Res. Pharmacol. Drug Discov..

[88] Rampen F.H.J. (1982). Malignant Melanoma: Sex Differences in Response to Chemotherapy?. Eur. J. Cancer Clin. Oncol..

[89] Lopes-Ramos C.M., Quackenbush J., DeMeo D.L. (2020). Genome-Wide Sex and Gender Differences in Cancer. Front. Oncol..

[90] Kamble S.M., Goyal S.N., Patil C.R. (2014). Multifunctional Pentacyclic Triterpenoids as Adjuvants in Cancer Chemotherapy: A Review. RSC Adv..

[91] Parikh K., Banna G., Liu S.V., Friedlaender A., Desai A., Subbiah V., Addeo A. (2022). Drugging KRAS: Current perspectives and state-of-art review. J. Hematol. Oncol..

