# Supplementary material for: The Triterpenoid CDDO-Methyl Ester Reduces Tumor Burden, Reprograms the Immune Microenvironment, and Protects from Chemotherapy-Induced Toxicity in a Preclinical Mouse Model of Established Lung Cancer
Source: Antioxidants (Basel). 2024 May 21;13(6):621. doi: 10.3390/antiox13060621 (PMC11201246; doi:10.3390/antiox13060621)
Supplement: Supplementary file 1 [file antioxidants-13-00621-s001.zip › antioxidants-2974454-supplementary.pdf]

Supplemental Figure 1

50mg/kg CDDO-Me

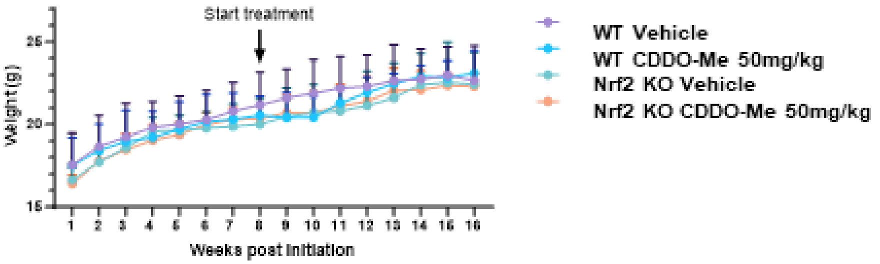

100mg/kg CDDO-Me

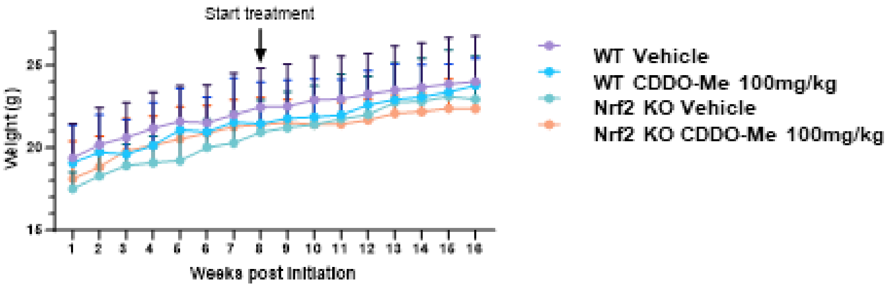

Supplemental Figure 1: Weights of wildtype (WT) and Nrf2 knockout (KO) mice treated with vehicle or 50-100mg/kg CDDO-Me for 8 weeks, as described in Fig 1.

Supplemental Figure 2

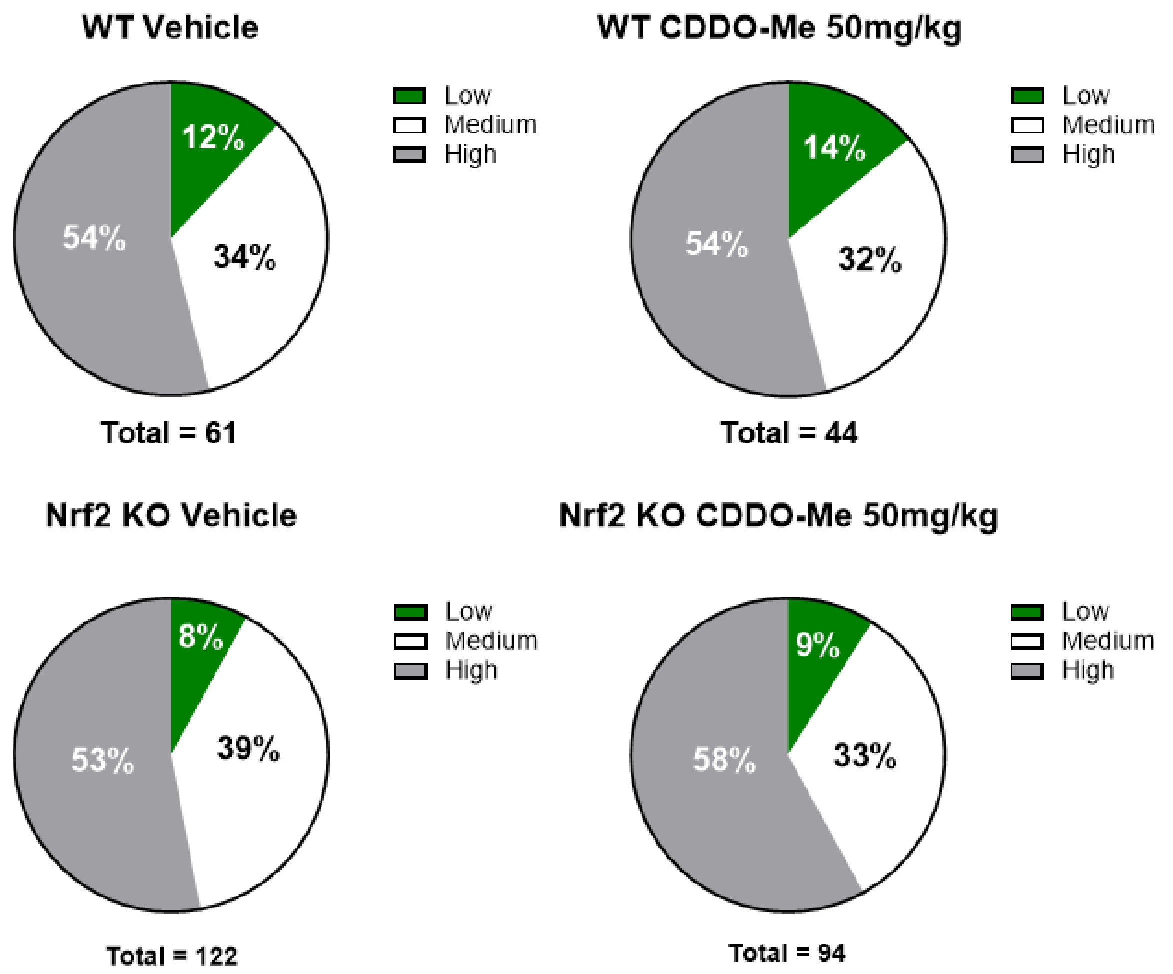

Supplemental Figure 2: Histopathological grades of lung tumors in WT and Nrf2 KO mice treated with vehicle and 50mg/kg CDDO-Me for 8 weeks, as described in Fig 1.

Supplemental Figure 3

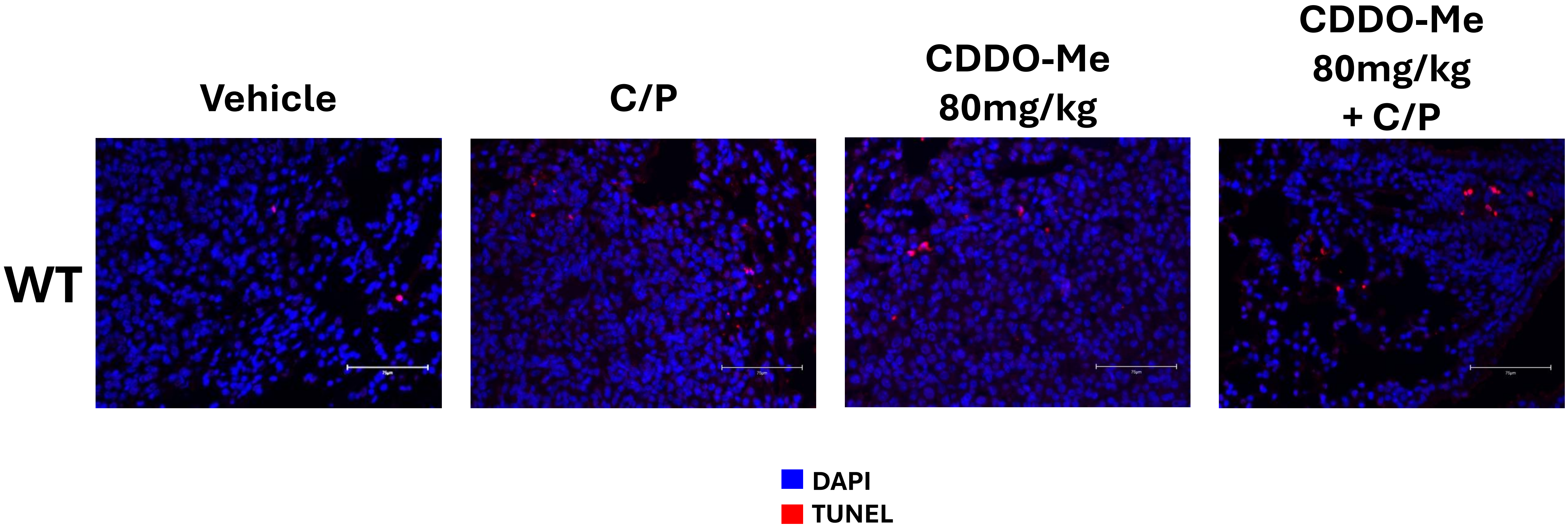

Supplemental Figure 3: Immunofluorescent staining for TUNEL in WT mice treated with vehicle, 80mg/kg CDDO-Me, carboplatin and paclitaxel (C/P), or the combination for 12 weeks, as described in Fig 3. TUNEL-positive cells were stained with Alexa Fluor 594 and nuclei were counterstained with DAPI.

Supplemental Figure 4

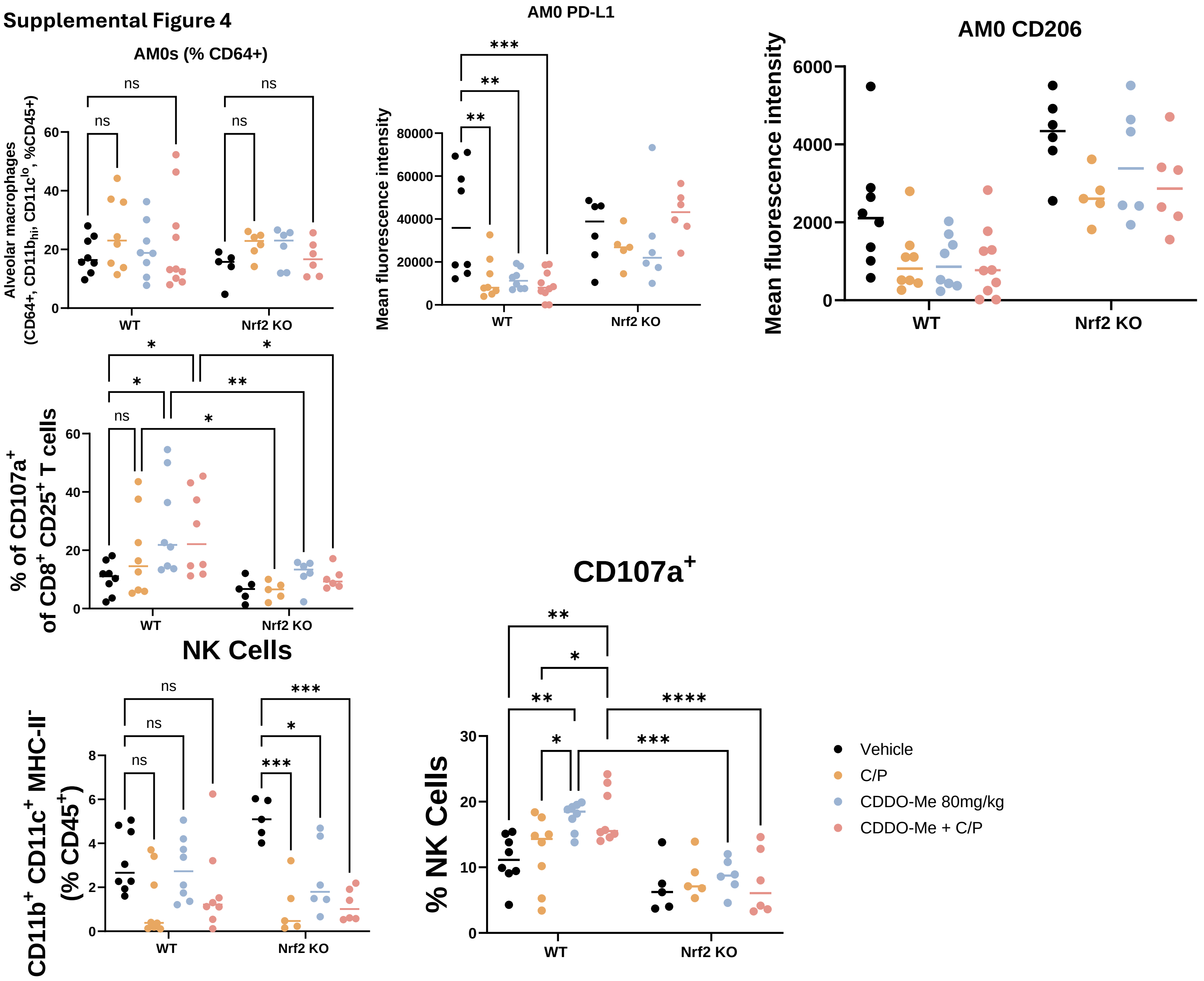

Supplemental Figure 4: Flow cytometric analysis of alveolar macrophages (CD64+ CD11b^{lo} CD11c^{hi}), CD8+ T cells, and NK cells (CD11c$^+$ CD11b^{lo} CD64$^+$, % CD45$^+$) in WT and Nrf2 KO mice treated with vehicle, 80mg/kg CDDO-Me, carboplatin and paclitaxel (C/P), or the combination for 12 weeks, as described in Fig 3. Two-way ANOVA followed by Tukey HSD. * $p < 0.05$; ** $p < 0.01$; *** $p < 0.001$; **** $p < 0.0001$

Supplemental Figure 5

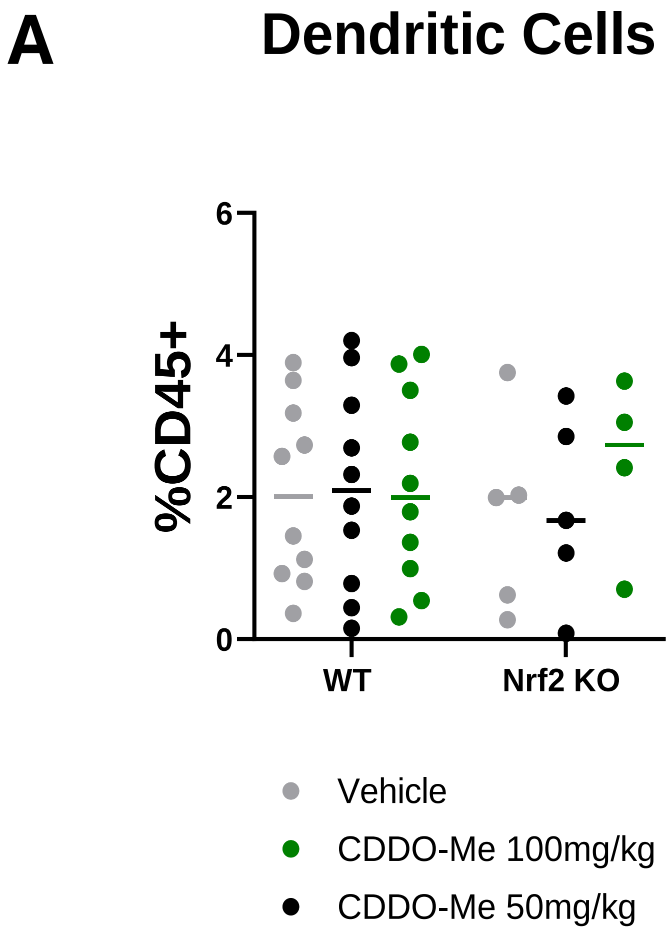

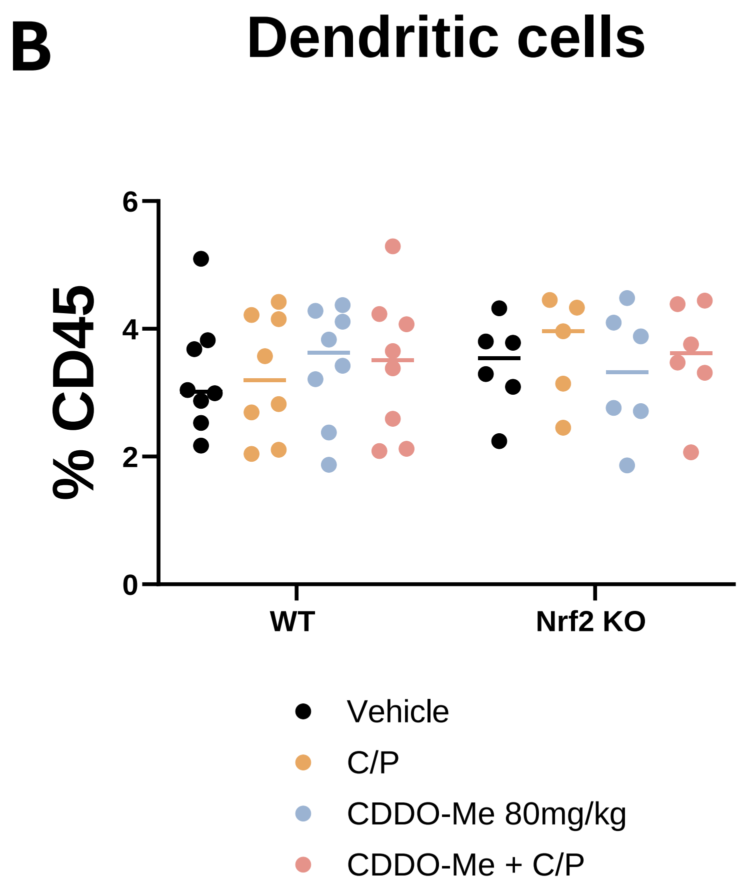

Supplemental Figure 5: **A**. Dendritic cells (CD45+, Gr1+, CD11b+, CD11c+, IA-IE+, CD24+; % CD45+) in the lungs of mice either treated with vehicle or CDDO-Me (50-100mg/kg). B. Dendritic cells (CD24+; % CD45+) in the lungs of mice either treated with vehicle or CDDO-Me (80mg/kg), ± C/P. Two-way ANOVA, no statistically significant differences.

Supplemental Figure 6

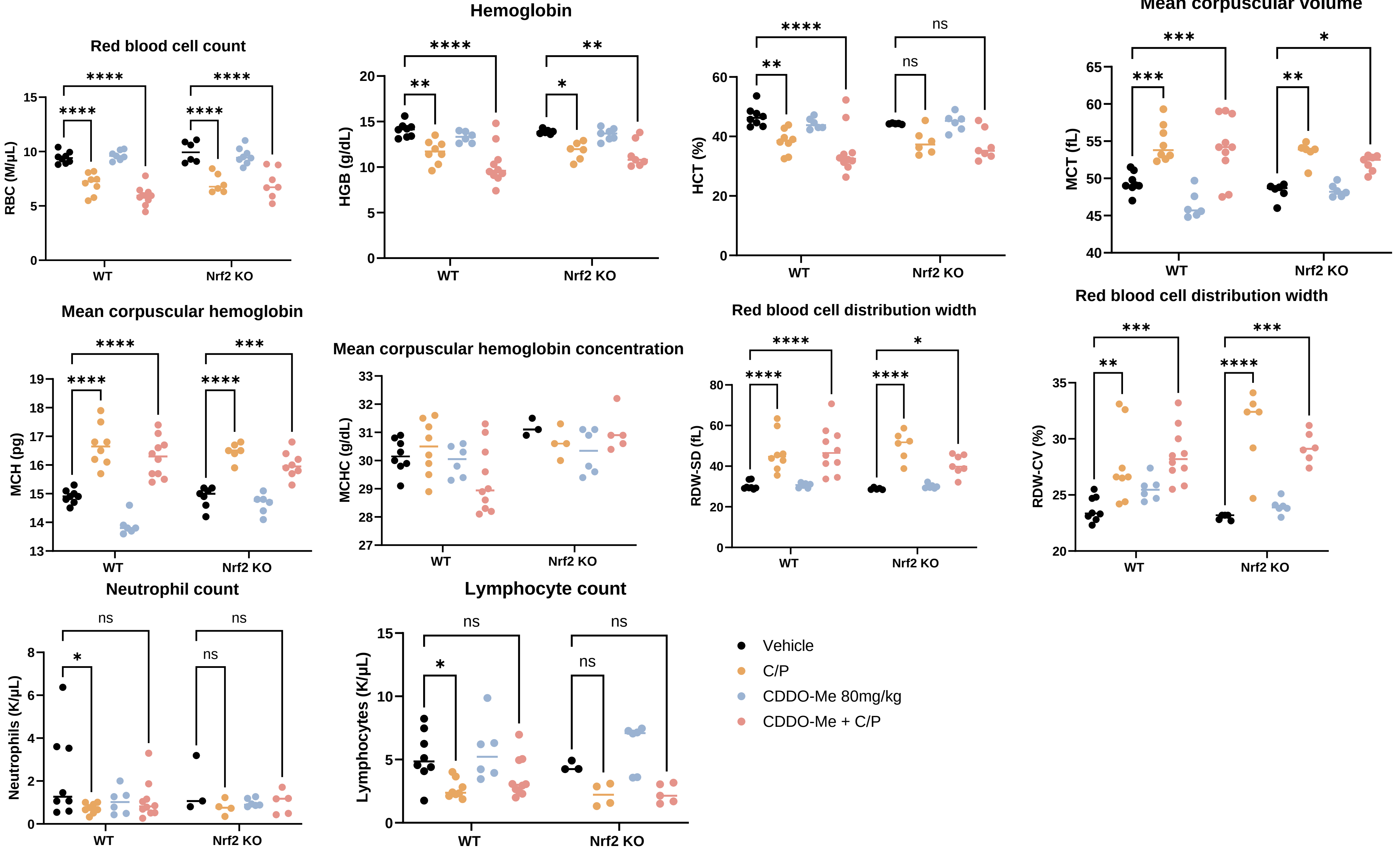

Supplemental Figure 6: Complete blood counts in WT and Nrf2 KO mice treated with vehicle, 80mg/kg CDDO-Me, carboplatin and paclitaxel (C/P), or the combination for 12 weeks, as described in Fig 3. Two-way ANOVA followed by Tukey HSD. * $p < 0.05$; ** $p < 0.01$; *** $p < 0.001$; **** $p < 0.0001$

Supplemental Figure 7

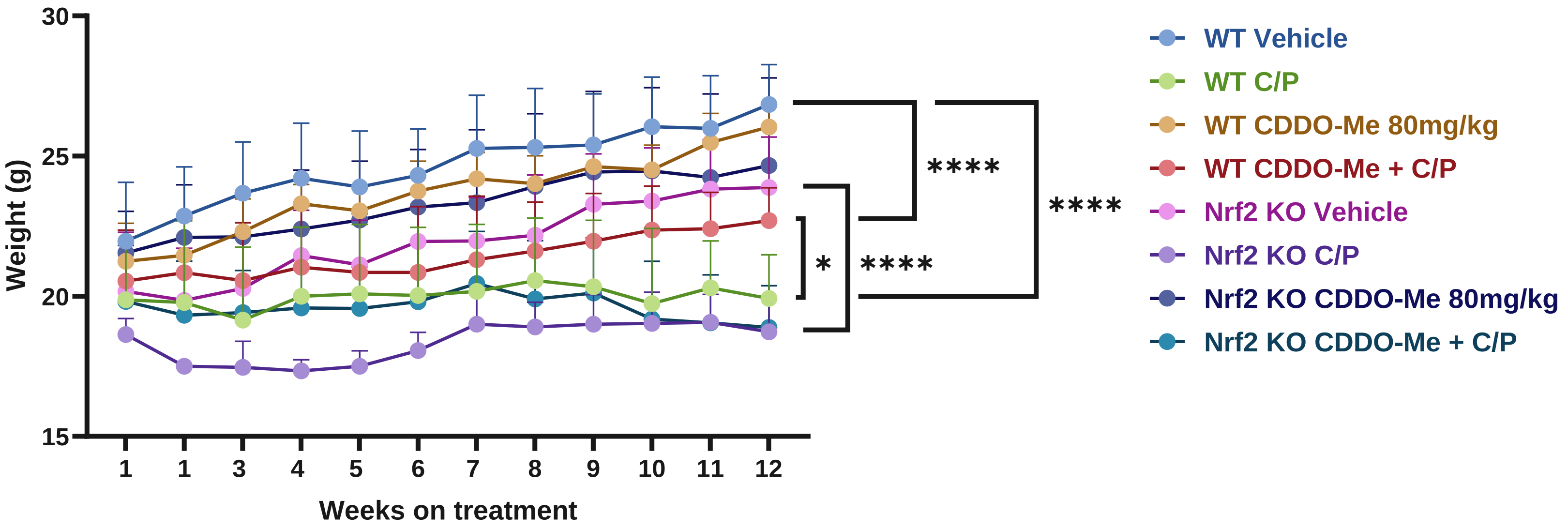

Supplemental Figure 7: Weights (g) measured once per week throughout the duration of the study of WT and Nrf2 KO mice treated with vehicle, 80mg/kg CDDO-Me, carboplatin and paclitaxel (C/P), or the combination for 12 weeks, as described in Fig 3. Two-way ANOVA followed by Tukey HSD at end time point (12 weeks). * $p < 0.05$; **** $p < 0.0001$.

Supplemental Table 1

	F WT Control	M WT Control	F WT CDDO-Me 50mg/kg	M WT CDDO-Me 50mg/kg	F Nrf2 KO Control	M Nrf2 KO Control	F Nrf2 KO CDDO-Me 50mg/kg	M Nrf2 KO CDDO-Me 50mg/kg
Surface tumors	168	184	119	112	431	381	418	438
Average per mouse (% WT control)	24 ± 1.2 (100%)	26.3 ± 1.5 (100%)	17 ± 1.1 (70.8%)	16 ± 1.4 (60.9%)	61.6 ± 2.4 (256.5%)	63.5 ± 3.0 (241.6%)	59.7 ± 3.1 (248.8%)	62.6 ± 3.3 (238%)
# of mice/group	7	7	7	7	7	6	7	7
Average # tumors/slide (% WT control)	2.29 ± 0.5 (100%)	2.07 ± 0.3 (100%)	1.64 ± 0.5 (71.9%)	1.5 ± 0.5 (72.4%)	3.79 ± 0.7 (165.6%) *	6.5 ± 0.5 (313.8%) **** #	3.64 ± 0.4 (159.4%) *	7.43 ± 0.8 (358.6%) **** #
Average Tumor Size (mm^3)/tumor (% WT control)	0.22 ± 0.1 (100%)	0.31 ± 0.1 (100%)	0.1 ± 0.07 (47%)	0.13 ± 0.09 (43.7%)	0.25 ± 0.1 (112.1%)	0.42 ± 0.2 (136%)	0.42 ± 0.1 (188.8%)	0.29 ± 0.2 (95.5%)
Average Tumor Burden (mm^3) (% WT control)	0.51 ± 0.1 (100%)	0.63 ± 0.2 (100%)	0.17 ± 0.07 (33.8%)	0.2 ± 0.02 (31.6%)	0.94 ± 0.2 (185.7%)	2.7 ± 0.5 (426.6%) **** #	1.52 ± 0.3 (300.9%)	2.17 ± 0.5 (342.6%) **** #
Low Grade (% total)	19	3.4 #	21.8	4.8 #	7.5	7.7	7.8	9.6
Medium Grade (% total)	43.5	24.2	30.4	33.3	45.3	34.6	31.4	33.7
High Grade (% total)	37.5	72.4 #	47.8	61.9	47.2	57.7	60.8	56.7

Supplemental Table 1: Number, size and histopathology of lung tumors in female (F) and male (M) WT and Nrf2 KO mice treated with vehicle and 50mg/kg CDDO-Me for 8 weeks, as described in Fig 1. Two-way ANOVA followed by Tukey HSD (tumor number, size, and burden); Z test for proportions (tumor histopathological grading): * $p < 0.05$ vs. WT control; **** $p < 0.0001$ vs. WT control; # $p < 0.05$ M vs. F

Supplemental Table 2

	F WT Control	M WT Control	F WT CDDO-Me 100mg/kg	M WT CDDO-Me 100mg/kg	F Nrf2 KO Control	M Nrf2 KO Control	F Nrf2 KO CDDO-Me 100mg/kg	M Nrf2 KO CDDO-Me 100mg/kg
Surface tumors	244	159	81	44	382	417	420	230
Average per mouse	22.2 ± 1.1	22.7 ± 1.4	7.4 ± 0.08	6.3 ± 0.09	54.6 ± 2.7	52.1 ± 3.0	52.5 ± 2.2	57.5 ± 2.9
% of control	100.0	100.0	33.2	27.7	246.0	229.5	236.7	253.1
# of mice/group	11	7	11	7	7	8	8	4
Average # tumors/slide (% F WT control)	1.8 ± 0.3 (100%)	3.2 ± 0.5 (181.3%) #	0.9 ± 0.3 (51.3%) *	0.2 ± 0.1 (12.1%) ****	3.2 ± 0.6 (181.3%) *	4.8 ± 0.6 (271.5%) * #	3.6 ± 0.4 (201%) *	4.6 ± 0.7 (260.9%) * #
Ave Tumor Size (mm^3)/tumor (% WT control)	0.13 ± 0.05 (100%)	0.2 ± 0.1 (100%)	0.07 ± 0.02 (52.4%)	0.06 ± 0.02 (27.5%)	0.24 ± 0.06 (177%)	0.32 ± 0.1 (159.3%)	0.26 ± 0.1 (193.4%)	0.26 ± 0.1 (128.5)
Ave Tumor Burden (mm^3) (% WT control)	0.24 ± 0.06 (100%)	0.66 ± 0.2 (100%) #	0.06 ± 0.02 (26.9%) *	0.01 ± 0.008 (1.8%) **	0.76 ± 0.2 (321%) *	1.56 ± 0.3 (238.5%) **** ###	0.91 ± 0.1 (388.6%) ****	1.21 ± 0.2 (184.9%) ****
Low Grade (% total)	20.5	8.9 #	40 *	0.0	6.7 *	11.5	8.8	10.8
Medium Grade (% total)	46.2	46.7	35	66.7	42.2	42.3	43.8	43.2
High Grade (% total)	33.3	44.4	25 *	33.3	51.1	46.2	47.4	46

Supplemental Table 2: Number, size and histopathology of lung tumors in female (F) and male (M) WT and Nrf2 KO mice treated with vehicle and 100mg/kg CDDO-Me for 8 weeks, as described in Fig 1. Two-way ANOVA followed by Tukey HSD (tumor number, size, and burden); Z test for proportions (tumor histopathological grading): * $p < 0.05$ vs. WT control; ** $p < 0.01$ vs. WT control; **** $p < 0.0001$ vs. WT control; # $p < 0.05$ M vs. F; ### $p < 0.001$ M vs. F

Supplemental Table 3

A

	F Nrf2 KO Control	M Nrf2 KO Control	F Nrf2 KO C/P	M Nrf2 KO C/P	F Nrf2 KO Me	M Nrf2 KO Me	F Nrf2 KO Me + C/P	M Nrf2 KO Me + C/P
Surface tumors	203	593	93	140	376	288	130	166
Average per mouse (% WT control)	67.7 ± 4.1 (176.3%)	98.8 ± 4.3 (238.2%)**	23.3 ± 2.3 (60.6%)	35 ± 2.5 (84.3%)*	62.7 ± 6.2 (163.3%)	96 ± 6.4 (231.3%)**	26 ± 3.9 (67.8%)	41.5 ± 4.1 (100%)*
# of mice/group	3.0	6.0	4.0	3.0	6.0	3.0	5.0	3.0
Average # tumors/slide (% WT control)	5.2 ± 0.6 (229.6%)	8.5 ± 1.0 (289.4%)	2.3 ± 0.6 (100%)	5.3 ± 0.9 (181.6%)	5.7 ± 0.5 (251.9%)	8.7 ± 0.7 (295%)	3.2 ± 0.6 (142.2%)	5.3 ± 0.8 (181.6%)
Average Tumor Size (mm^3)/tumor (% WT control)	0.5 ± 0.6 (155.1%)	1.1 ± 0.8 (234%)	0.2 ± 0.2 (46.9%)	0.2 ± 0.3 (42.7%)	0.7 ± 0.3 (196.9%)	1.1 ± 0.8 (226.4%)	0.1 ± 0.1 (37.8%)	0.3 ± 0.2 (71%)
Average Tumor Burden (mm^3) (% WT control)	2.8 ± 0.3 (356.1%)	9.4 ± 0.5 (677.2%)****	0.4 ± 0.1 (46.9%)	1.1 ± 0.1 (77.5%)	3.9 ± 0.1 (496%)	9.2 ± 0.6 (667.9%)***	0.4 ± 0.08 (53.8%)	1.8 ± 0.2 (128.9%)
Low Grade (% total)	3.2	1.0	27.8	15.6*	1.5	0.0	28.1	15.6*
Medium Grade (% total)	22.6	14.7	61.1	37.5*	20.6	19.2	46.9	34.4
High Grade (% total)	74.2	84.3	11.1	46.9*	77.9	80.8	25.0	50*

B

	F WT Control	M WT Control	F WT C/P	M WT C/P	F WT CDDO-Me 80mg/kg	M WT CDDO-Me 80mg/kg	F WT CDDO-Me + C/P	M WT CDDO-Me + C/P
Surface tumors	307	332	151	76	132	99	41	34
Average per mouse (% WT control)	38.4 ± 1.6 (100%)	41.5 ± 1.9 (100%)	18.875 ± 1.4 (49.2%)	19 ± 1.8 (45.8%)	18.9 ± 1.2 (49.1%)	33 ± 1.4 (79.5%)*	5.1 ± 1.1 (13.4%)	6.8 ± 1.3 (16.4%)
# of mice/group	8.0	8.0	8.0	4.0	7.0	3.0	8.0	5.0
Average # tumors/slide (% WT control)	2.3 ± 0.4 (100%)	2.9 ± 0.4 (100%)	1.6 ± 0.2 (69.4%)	1.9 ± 0.2 (63.8%)	2.1 ± 0.3 (92.1%)	2.7 ± 0.7 (90.8%)	1.2 ± 0.3 (52.8%)	1.7 ± 0.4 (57.9%)
Average Tumor Size (mm^3)/tumor (% WT control)	0.4 ± 0.2 (100%)	0.5 ± 0.3 (100%)	0.09 ± 0.02 (24.3%)	0.2 ± 0.1 (38.7%)	0.1 ± 0.05 (41%)	0.2 ± 0.1 (43.8%)	0.03 ± 0.02 (9.8%)	0.07 ± 0.04 (15.2%)
Average Tumor Burden (mm^3) (% WT control)	0.8 ± 0.4 (100%)	1.4 ± 0.2 (100%)	0.1 ± 0.03 (16.9%)	0.3 ± 0.1 (24.7%)	0.3 ± 0.04 (37.7%)	0.5 ± 0.1 (39.8%)	0.04 ± 0.02 (5.2%)	0.1 ± 0.04 (8.8%)
Low Grade (% total)	5.6	0.0	20.0	13.3	6.9	6.2	57.9	23.5*
Medium Grade (% total)	22.2	23.4	36.0	46.7	31.0	31.3	31.6	47.1
High Grade (% total)	72.2	76.6	44.0	40.0	62.1	62.5	10.5	29.4*

Supplemental Table 3: Number, size and histopathology of lung tumors in female (F) and male (M) WT **(A)** and Nrf2 KO **(B)** mice treated with vehicle, 80mg/kg CDDO-Me, carboplatin and paclitaxel (P/C), or the combination for 12 weeks, as described in Fig 3. Two-way ANOVA followed by Tukey HSD (tumor number, size, and burden); Z test for proportions (tumor histopathological grading): * $p < 0.05$ M vs. F; ** $p < 0.01$ M vs. F; *** $p < 0.001$ M vs. F; **** $p < 0.0001$ M vs. F.
